# Arbuscular Mycorrhizal Symbiosis Triggers Major Changes in Primary Metabolism Together With Modification of Defense Responses and Signaling in Both Roots and Leaves of *Vitis vinifera*

**DOI:** 10.3389/fpls.2021.721614

**Published:** 2021-08-25

**Authors:** Mary-Lorène Goddard, Lorène Belval, Isabelle R. Martin, Lucie Roth, Hélène Laloue, Laurence Deglène-Benbrahim, Laure Valat, Christophe Bertsch, Julie Chong

**Affiliations:** ^1^Laboratoire Vigne, Biotechnologies et Environnement (LVBE, UPR 3991), Université de Haute Alsace, Colmar, France; ^2^Laboratoire d'Innovation Moléculaire et Applications, Université de Haute-Alsace, Université de Strasbourg, CNRS, LIMA, UMR 7042, Mulhouse, France

**Keywords:** arbuscular mycorrhiza, grapevine, metabolomics, defense responses, metabolites

## Abstract

Grapevine (*Vitis vinifera* L.) is one of the most important crops worldwide but is subjected to multiple biotic and abiotic stresses, especially related to climate change. In this context, the grapevine culture could take advantage of symbiosis through association with arbuscular mycorrhizal fungi (AMF), which are able to establish symbiosis with most terrestrial plants. Indeed, it is well established that mycorrhization improves grapevine nutrition and resistance to stresses, especially water stress and resistance to root pathogens. Thus, it appears essential to understand the effect of mycorrhization on grapevine metabolism and defense responses. In this study, we combined a non-targeted metabolomic approach and a targeted transcriptomic study to analyze changes induced in both the roots and leaves of *V. vinifera* cv. Gewurztraminer by colonization with *Rhizophagus irregularis* (Ri). We showed that colonization of grapevine with AMF triggers major reprogramming of primary metabolism in the roots, especially sugar and fatty acid metabolism. On the other hand, mycorrhizal roots had decreased contents of most sugars and sugar acids. A significant increase in several fatty acids (C16:1, linoleic and linolenic acids and the C20 arachidonic and eicosapentaenoic acids) was also detected. However, a downregulation of the JA biosynthesis pathway was evidenced. We also found strong induction of the expression of PR proteins from the proteinase inhibitor (PR6) and subtilase (PR7) families in roots, suggesting that these proteins are involved in the mycorrhiza development but could also confer higher resistance to root pathogens. Metabolic changes induced by mycorrhization were less marked in leaves but involved higher levels of linoleic and linolenic acids and decreased sucrose, quinic, and shikimic acid contents. In addition, Ri colonization resulted in enhanced JA and SA levels in leaves. Overall, this study provides a detailed picture of metabolic changes induced by AMF colonization in a woody, economically important species. Moreover, stimulation of fatty acid biosynthesis and PR protein expression in roots and enhanced defense hormone contents in leaves establish first insight in favor of better resistance of grapevine to various pathogens provided by AMF colonization.

## Introduction

Vine and wine are very ancient and represent both a part of human history and a significant socioeconomic sector (Reynolds, 2017). Today, grapevine is a very important crop worldwide, with a global production area of 6.8 Mha in 2018, generating a global value of 31.4 billion euros for world wine trade (OIV, 2018). In the context of global warming, grapevine culture, like other cultivated plants, is confronted with multiple stresses, especially heat stress and drought (Songy et al., 2019). In addition to reduced growth and yield, it is well known that these stresses will lead to the weakening of the plant that could explain the recent explosion of grapevine dieback syndromes such as trunk diseases.

In order to gain better resistance to multiple stresses, the grapevine culture could take advantage of symbiosis through association with arbuscular mycorrhizal fungi (AMF). AMF belong to the *Glomeromycota* phylum and are soil microorganisms that are able to establish symbiosis with most terrestrial plants (Trouvelot et al., 2015). In this mutualistic association, a plant supplies fungi with carbohydrates and lipids, and fungal colonization increases the root absorption surface, thus improving plant access to water and minerals. These exchanges take place in root cortical cells where specialized structures called “arbuscules” are developed.

It is well established that association with AMF reduces the need of fertilizers in plants by enhancing the uptake of nutrients such as nitrogen and phosphate (Trouvelot et al., 2015). In grapevines, it has also been recently demonstrated that mycorrhization with *Funneliformis mosseae* (*F. mosseae*) increases shoot growth and root system branching of acclimatized plantlets, correlated with high expression of two grapevine phosphate transporters (Valat et al., 2018). It is also known that AMF association can improve plant tolerance to abiotic stresses. In grapevines, colonization with AMF could result in more efficient water uptake and better resistance to water stress (Trouvelot et al., 2015). Indeed, higher mycorrhizal colonization was evidenced in drier soil areas (Donkó et al., 2014). In addition, colonization of 1-year-old grapevines by AMF increased the water flux and photosynthesis compared to non-colonized plants (Nogales et al., 2020). In the same study, Nogales et al. (2020) further investigated responses of mycorrhized cv. Touriga Nacional grapevines to heat stress. They reported that colonization with *Rhizophagus irregularis* (*R. irregularis*) could help the plant to sustain its growth after heat shocks. In addition to water stress tolerance, AMF symbiosis has the potential to improve grapevine tolerance to salinity and heavy metals (Trouvelot et al., 2015).

Interestingly, it has been reported that several plant–AMF associations also result in better resistance to biotic stresses. Resistance mechanisms involve both competition for colonization sites and induction of plant defense responses. Indeed, root inoculation with AMF triggers the so-called “mycorrhiza-induced-resistance” (MIR), which is systemic protection against a wide range of pathogens (Cameron et al., 2013). Although MIR has been shown to be efficient against a wide range of diseases in diverse plants, this kind of resistance has mainly been involved in the case of root diseases in grapevines (Petit and Gubler, 2006; Nogales et al., 2009; Hao et al., 2012). Concerning resistance to foliar diseases, Bruisson et al. (2016) showed that mycorrhization of several grapevine varieties (Chasselas, Pinot Noir, and the interspecific hybrid Divico) with *R. irregularis* triggered a higher expression of genes involved in stilbene biosynthesis; a higher bioactive stilbene content after leaf inoculation with *Botrytis cinerea* (*B. cinerea*) or *Plasmopara viticola* (*P. viticola*) was also recorded. Priming of defense reactions in leaves induced by AMF root colonization, therefore, supports the hypothesis of a role of mycorrhization in improving resistance to foliar diseases in grapevines. In a recent study, Cruz-Silva et al. (2021) have further revealed that mycorrhization of *Vitis vinifera* cv. Cabernet Sauvignon with *R. irregularis* altered the expression of several *P. viticola* effectors after infection, suggesting that AMF could enhance resistance to downy mildew.

Regarding all the beneficial effects provided by AMF associations, understanding the effect of mycorrhization on grapevine metabolism appears essential. Indeed, it is known that metabolic status is crucial to plant resistance to both abiotic and biotic stresses. In the Tempranillo variety, which is widely cultivated in Mediterranean areas, mycorrhizal symbiosis with a commercial inoculum of five AMFs triggered primary metabolite profile alterations in berries, with an increase in glucose and amino acids (Torres et al., 2019). Torres et al. (2018a) also found that AMF colonization of Tempranillo increased anthocyanin contents in the berries of fruit cuttings and modulated ABA metabolism, especially under high temperatures. Still, in Tempranillo grapevines, mycorrhization with a mixture of five AMFs induced the accumulation of phenolics, hydroxycinnamic acids, and carotenoids in leaves, although with different levels depending on the clone analyzed (Torres et al., 2018b). Finally, in recent studies, a significant increase in volatile organic compounds was reported in *V. vinifera* Cabernet Sauvignon roots (Velásquez et al., 2020a) and *V. vinifera* Sangiovese leaves (Velásquez et al., 2020b) after colonization with *F. mosseae*. These VOCs included terpenes and monoterpene alcohols related to plant defense (Velásquez et al., 2020a).

Few studies have focused on both transcriptomic and metabolomic changes induced by mycorrhization in roots and leaves of grapevines. Balestrini et al. (2017) compared the effect of a single AMF (*F. mosseae*) and of a mixed inoculum containing bacteria and fungi on the transcriptome reprogramming of 110R rootstock roots. Although the mixed inoculum elicited a more important transcriptional regulation, the expression of genes involved in nutrient transport, transcription factors, and cell wall-related genes was significantly altered in the two conditions. In this study, in order to better understand the effect of mycorrhization on grapevine metabolism and defense reactions, we combined a non-targeted metabolomic approach and a targeted transcriptomic study to analyze changes induced in both the roots and leaves of *V. vinifera* cv. Gewurztraminer following colonization with *R. irregularis* DAOM 197198. This AMF strain has been sequenced and is commercially available for winegrowers. The non-targeted metabolomic study used GC-MS and LC-MS to highlight the effect of Ri colonization on root and leaf primary and specialized metabolites. The consequence of mycorrhization was further analyzed by targeted analysis of the contents in defense hormones jasmonates and salicylic acid and by the study of the expression of genes involved in defense responses, sugar transport and metabolism, and jasmonic acid biosynthesis.

## Materials and Methods

### Plant Culture and Mycorrhization

Two-node segments of *V. vinifera* (L.) cv. Gewurztraminer clone 643 were collected in the winter of 2018–2019 in the experimental vineyard from INRAE (Colmar, France), treated with Beltanol-L, and briefly conserved at 4°C. Wood cuttings were previously dipped with indole-3-butyric acid (IBA, 1 mg/ml, Sigma Aldrich, MO, USA) and then planted in individual 1-L pots (four cuttings/pot) for rooting in a 1:1 sterile mix of sand and perlite. The pots were then placed in a growth chamber at 27°C with a 16 h photoperiod (150 μEm^−2^·s^−1^ light irradiance, 25/20°C day/night). Plants were watered to saturation two times a week with tap water for 3 weeks; then, with a low Pi nutritive solution [2.5 mM Ca(NO_3_)_2_, 2.5 mM KNO_3_, 0.1 mM KH_2_PO_4_, 1 mM MgSO_4_, 50 μM EDTA-Fe(III)-Na, 10 μM H_3_BO_3_, 2 μM MnCl_2_, 1 μM ZnSO_4_, 0.5 μMCuSO_4_, and 0.05 μM Na_2_MoO_4_]. After 5–6 weeks of culture, well-rooted plants with homogenous growth were transplanted individually (1 plant per pot) in 1 L pots containing a 1:1 sterile mix of sand and perlite. Plants were watered to saturation two times a week, alternately with tap water and low Pi solution. After 10 days, one batch of 12 plants was inoculated with *R. irregularis* DAOM197198 (Ri) produced under axenic conditions (Agronutrition, Carbonne, France): the Ri suspension was diluted with tap water to contain 50 spores/ml and 1,000 spores (20 ml) were gently poured at the base of the plant stem. Plants of the non-inoculated control plant batch (14 plants) were reated with 20 ml of diluted spore conservation solution without spores. Two months after mycorrhization, the plants were carefully removed from the culture pots. Root systems were separated from the aerial parts and rinsed in tap water. Fully expanded leaves, full length roots, and root tips were harvested and snap frozen in liquid N_2_ for transcriptomic and metabolomic analysis.

### Evaluation of AMF Colonization

Non-lignified roots (~1 g) were randomly harvested from each plant and stored in a lactoglycerol solution (lactic acid/glycerol/demineralized H_2_O 1:1:1) at 4°C. For coloration, roots were put in 15-ml polypropylene tubes and cleared for 20 min at 90°C in 10% (w/v) KOH, and then five droplets of H_2_O_2_ solution (30% v/v) were added under agitation. Roots were subsequently rinsed gently two times with demineralized water, and then immersed in an ink solution (5% Black Schaeffer Skrit ink/8% acetic acid) for 5 min at 90°C. Roots were then rinsed three times with demineralized water and discolored with acetic acid (8%) for 15 min at room temperature. After washing with demineralized water, the samples were stored in lactoglycerol at 4°C. Thirty fragments, ca. 10 mm long, were randomly collected from each sample, mounted 10 per slide in glycerol, and observed at × 200 magnification. Mycorrhization efficiency parameters were evaluated according to Trouvelot et al. (1986): frequency (F): percentage of colonized fragments, intensity (M): estimation of the proportion of hyphae in the root cortex, (a): arbuscule abundance in the mycorrhized root part, and (A): arbuscule abundance in the whole root system.

### Transcriptomic Analysis by RT-qPCR

Leaf and root tip samples were finely grinded in liquid nitrogen. RNA extraction and DNase I treatment were performed on root tips and fully expanded leaves as described by Valat et al. (2018). Reverse transcription was performed on 1 μg of RNA with the iScript™ Reverse Transcription Supermix (Biorad, France). For real-time PCR, reactions were carried out on the CFX96 system (Biorad, France). PCR reactions were carried out in triplicates in a reaction buffer containing 1X iQ SYBR^®^ Green Supermix, 0.2 mM of forward and reverse primers, and 10 ng of reverse transcribed RNA in a final volume of 25 μl. Thermal cycling conditions were 30 s at 95°C, followed by 40 cycles of 15 s at 94°C, 30 s at 60°C, and 30s at 72°C. The specificity of the individual PCR amplification was checked using a heat dissociation curve from 55 to 95°C following the final cycle of the PCR and by sequencing the final PCR products. The results obtained for each gene of interest were normalized to the expression of two reference genes (*VvEF1*α and *VvActin*). Relative expression (2^−ΔΔCT^) compared to the sample with the lowest expression (highest CT value) was also calculated as described in Schmittgen and Livak (2008). Mean values and standard deviations were obtained from 3 technical and 12 biological replicates. Primers used for real-time quantitative PCR are listed in Supplementary Table 1. Preliminary analysis of *PR* gene expression was performed with the NeoViGen96 chip on three independent biological replicates for each condition as described in Dufour et al. (2016).

### Metabolomic Analysis

#### Extraction

We analyzed 14 independent biological replicates for the NM condition and 11 independent biological replicates for the Ri condition. Leaves and whole roots were ground in liquid nitrogen and freeze-dried. About 10 and 5 mg of these respective tissues were precisely weighted and transferred to 1.5 ml polypropylene microtubes for two successive extractions of primary and specialized metabolites from the same starting material. For primary metabolite extraction, the samples were extracted with 600 μl for leaves and 300 μl for roots of extract solution composed of 50 mM potassium phosphate buffer pH 6 and 10 mg/L of phenoxyacetic acid as an internal standard in an ultrasound bath during 30 min (power 9, 20°C as starting temperature). After centrifugation (20,000 g, 20 min, 20°C), supernatants were transferred to a filtration plate (96-well plates Acroprep 1 ml, 45 μm GHP membrane, Pall Life Science, Portsmouth, United Kingdom) and filtered under a vacuum with a vacuum manifold (Pall Corporation, Portsmouth, United Kingdom) to be collected on a collector plate (a 96-well PP plate, 1.2 ml, VWR 732-2890/391-0077, Fontenay-sous-Bois, France). This operation was performed two times for each sample, and filtrates were gathered in the same proportions prior to GC-MS analyses.

After primary metabolite extraction, the remaining pellet was washed with ultrapure water, freeze-dried, and re-lyophilized for specialized metabolite extraction. The second extraction was carried out in the same conditions but with 300 μl of LC-MS grade methanol (Fisher Scientific, Illkirch, France), containing 5-methylsalicylic acid as an internal standard at 5 mg/L for both leaves and roots. After two extractions, filtrates were equally pooled and analyzed by LC-MS. The methods GC-MS and LC-MS have been described in detail in Labois et al. (2020).

#### GC-MS Analysis

GC-MS analysis was carried out on 30 μl of each aqueous extract after freeze-drying and derivatization reaction, which consists of subsequent addition of methoxyamine hydrochloride solution (20 μl, 30 mg/ml in anhydrous pyridine) followed by MSTFA (80 μl). The samples were respectively incubated during 90 and 30 min at 37°C and 600 rpm.

GC-MS analysis was performed on GC-2010 gas chromatography, coupled with the GC-QP2010 mass detector (Shimadzu Corporation, Tokyo, Japan) in the acquisition conditions already described by Labois et al. (2020).

After data file conversion, thanks to the ABF file converter (Reifycs), raw data were processed using MS-DIAL 4.0 (version 4.48) software (http://prime.psc.riken.jp/) for peak detection and integration and sample alignment. Identification of compounds was performed, thanks to an owner commercial compound library and the mass spectral library NIST 17 by comparison of retention time and a spectrum profile.

#### HPLC-MS Analysis

Methanolic extracts were analyzed on the High-Performance Liquid Chromatography Agilent 1,100 series coupled to the Agilent 6,510 accurate-mass Quadrupole-Time of Flight (Q-TOF) mass spectrometer with an electrospray ionization (ESI) interface in a negative ionization mode (Agilent Technologies, Santa Clara, CA, USA). Separation and detection of compounds were performed as described in Labois et al. (2020). Deconvolution, integration, and alignment were performed, thanks to the Profinder software (version B.08.00, Agilent Technologies, Santa Clara, CA, USA). Compound annotation was carried out with commercially available standards. Additional compounds were putatively identified using the molecular formula calculated from the exact mass and an isotope profile, relative retention times, and mass spectra from Metlin (https://metlin.scripps.edu).

### Extraction and Quantitative HPLC-MS Analysis of Jasmonates and Salicylic Acid

Fresh leaves and whole roots were manually ground in liquid nitrogen. Extraction and quantification of jasmonates were performed as described in Widemann et al. (2013) and Smirnova et al. (2017). Approximately 90 mg of powder, exactly weighted, was extracted with 900 μl of extraction solution (methanol: water: acetic acid (70:29:0.5), containing two internal standards: 9,10-dihydrojasmonoylisoleucine (dihydro-JA-Ile, 2 μM) and prostaglandine A1 (PGA1, 50 nM). The samples were agitated on a wheel for 30 min at 4°C and then centrifuged at maximum speed for 15 min at 4°C. About 450 μl of supernatant was transferred in a 1.5-ml microtube and concentrated to 250 μl in a speed vac at 30°C. The samples were stored overnight at −20°C and then centrifuged for 10 min at maximal speed to pellet insoluble material. Approximately 150 μl of the particle-free supernatant was transferred to an amber glass vial to be injected in LC-MS/MS.

Jasmonates [jasmonic acid (JA), jasmonoyl-isoleucine (JA-Ile), 12-oxophytodienoic acid (12-OPDA)] and salicylic acid (SA) were identified and quantified with a UHPLC system Elute connected to a mass spectrometer Impact II Q-TOF-MS/MS equipped with an electrospray ESI source Apollo II operating in a negative mode (Bruker Daltonics GmbH, Bremen, Germany). The raw samples were separated, thanks to the reversed-phase C18 Bruker Intensity Solo HPLC column (2-μm particle size, 100 Å pore size, and of 2 × 100-mm dimensions) (BRHSC18022100, Bruker Daltonics GmbH, Bremen, Germany). The mobile phases were ultrapure water with 0.1% formic acid (eluent A) and MeOH with 0.1% formic acid (eluent B). They were used in a gradient expressed as B percentage: 2 min at 1.%, a linear gradient during 15 min until 99%, 3 min at 99%, followed by 5 min in the initial conditions (1% B) for equilibration before a new injection. The total flow rate was 0.25 ml/min, injection volume was 10 μl, and the oven column and the autosampler temperature were 35 and 8°C, respectively. The MS detector was internally calibrated before starting the analysis batch and additionally at the beginning of each injection by infusing a 10-mM sodium formate solution in iPrOH: H_2_O (1:1, v/v). The ESI parameters were as follows: capillary voltage, −3,500 V; end-plate offset, −500 V; nebulizer gas, 29 psi; dry gas, 8 L/min; and dry temperature, 200°C. The spectra rate was 2 Hz over a 30–1,000 m/z mass range of both auto MS/MS and bbCID scan modes. To determine fragments of each parent metabolite, MS/MS spectra were recorded with a collision energy ramp from 20 to 50 eV. In the broadband collision-induced dissociation (bbCID) mode, each parent ion was fragmented by alternating low- and high-collision energy, namely, 6 and 50 eV, respectively. Data acquisition was achieved with the Bruker Compass HyStar 5.1 and otofControl 5.2 software, and data treatment was performed with the DataAnalysis 5.3 and TASQ 2021 (Bruker Daltonics GmbH, Bremen, Germany).

Qualifier ions were determined from data obtained in an auto MS/MS acquisition mode in DataAnalysis and listed in Supplementary Table 2 together with chromatographic retention times, assigned internal standards, and quantifier ions to achieve quantification in TASQ. Absolute quantification was carried out, thanks to external calibration from commercially available standards (serial dilutions were performed from a 200 nM stock solution (SA and PGA1) and a 100 nM stock solution (12-OPDA, JA, JA-Ile, dh-JA-Ile) in methanol. Calibrants were analyzed in triplicates in the same acquisition method as samples. Final results were given in nanogram of each compound related to gram of fresh weight raw material after normalization by the internal standard and fresh weight of raw material.

### Statistical Analysis

For all GC-MS and LC-MS data, the area of each compound was normalized with respect to the raw material weight and the internal standard area. Only metabolites present in 80% of the samples of each condition were considered. Statistical analyses were performed with the MetaboAnalyst 5.0 online platform (https://www.metaboanalyst.ca (Chong et al., 2019). Normalized areas were transformed using the base 10 logarithm and mean-centering. Metabolites significantly different between NM and Ri conditions were identified with a fold change of at least +/– 1.5 and a p value < 0.05 (Wilcoxon test analysis).

Transcriptomic data were analyzed using a non-parametric test (Kruskall and Wallis) with the R 4.0.0 software.

## Results

### Plant Growth Parameters and Analysis of Mycorrhization

**Figure 1:**
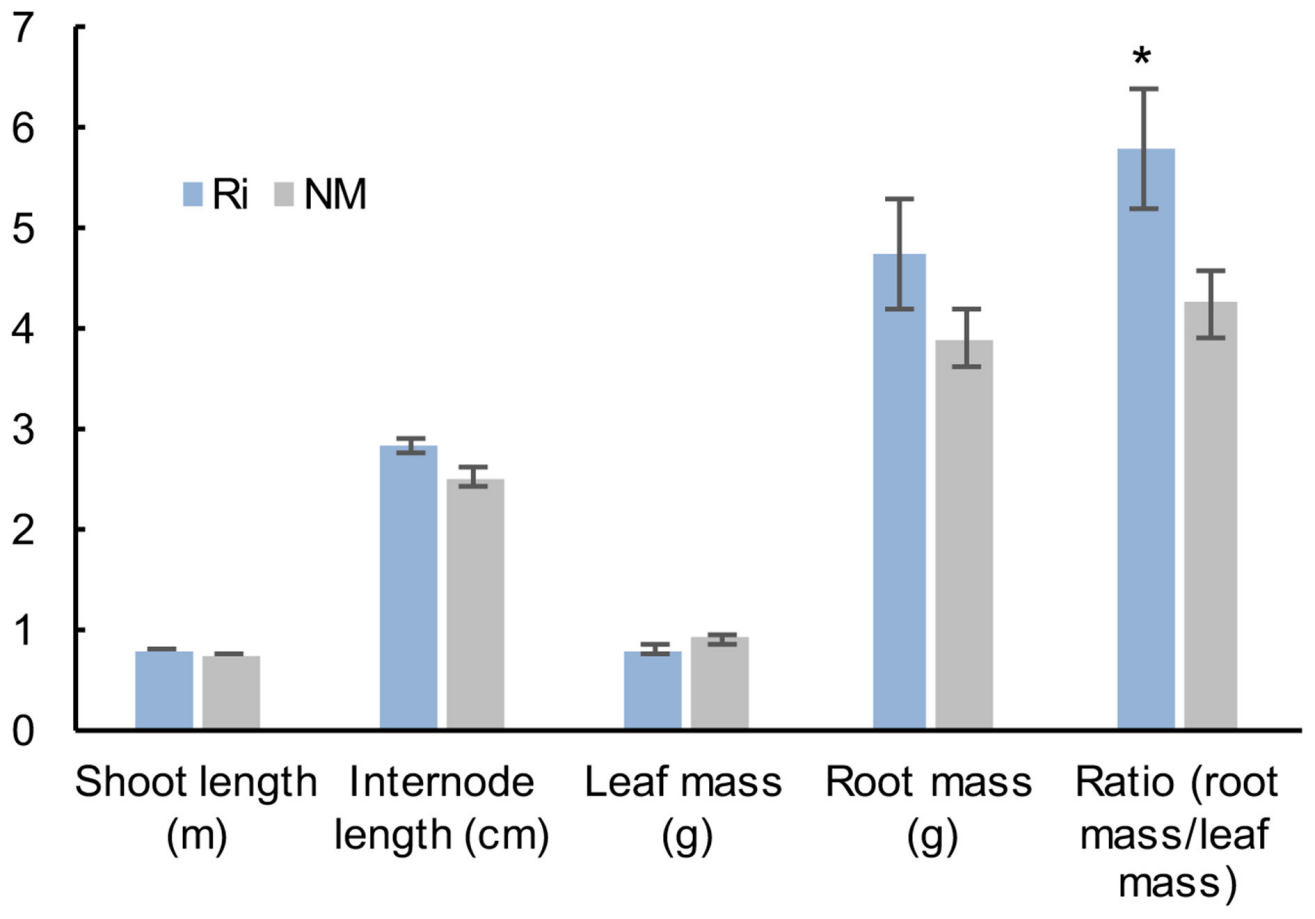
Plant growth parameters of grapevine wood cuttings inoculated (Ri) or not (NM) with *R. irregularis*. Shoot length, internode length, mass of the seven first leaves, and whole root system mass (fresh weights) were measured 2 months after mycorrhization. Results are means ± SE of 12 biological replicates. Asterisks indicate significant difference between NM and Ri condition (Kruskall and Wallis, *p* < 0.05).

Analysis of plant growth parameters 2 months after mycorrhization showed that shoot length, internode length, and leaf mass were not significantly different between non-mycorrhized (NM) and mycorrhized (Ri) plants. However, Ri plants were characterized by a higher mass of the root system (although not statistically significant) as well as a higher root mass/leaf mass ratio (Figure 1).

**Figure 2:**
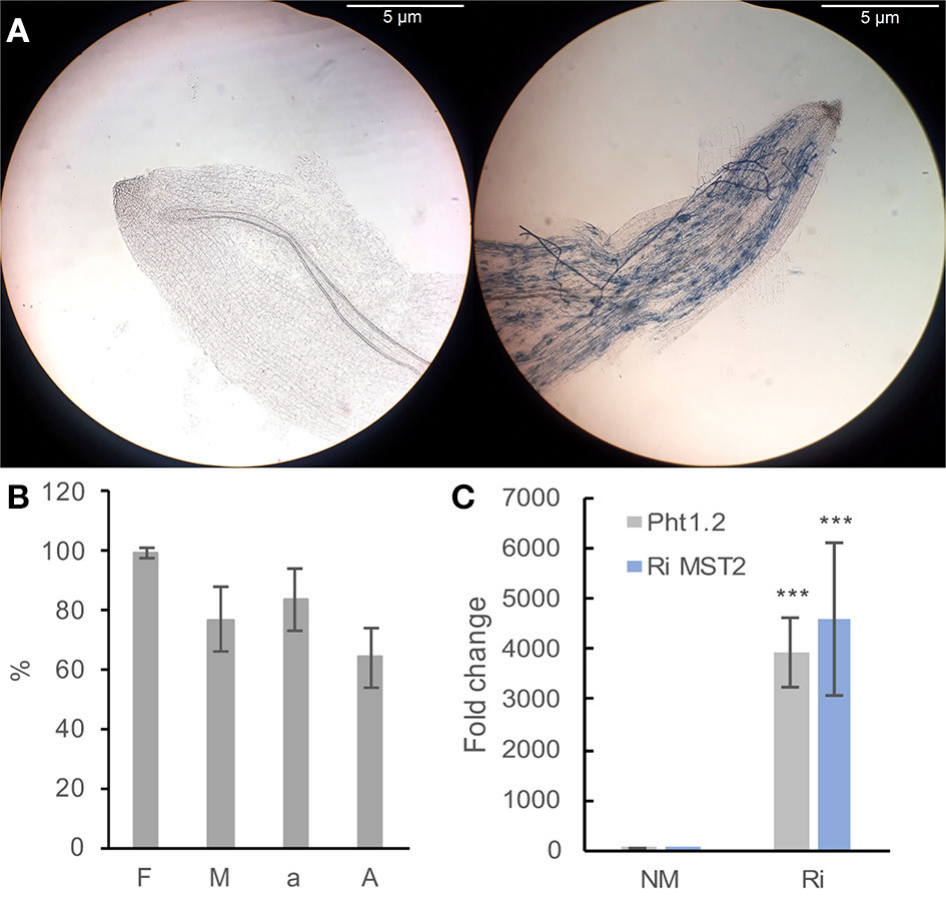
Plant mycorrhization parameters **(A,B)** and expression of colonization marker genes in grapevine roots **(B)**. **(A)** Representative pictures of Ri (right) and NM (left) roots. Pictures were taken with a Nikon ALPHAPHOT-2 microscope (X100 magnification). Bar = 5 μm. **(B)** F (frequency): percentage of colonized fragments, M (intensity): proportion of hyphae in the root cortex, a: arbuscule content in colonized roots, A: arbuscule abundance in the whole root system (from Trouvelot et al., 1986). Data are the mean ± SD of 12 biological replicates. **(C)** Expression of the grapevine *Pht1.2* phosphate transporter and the *R. irregularis RiMST2* hexose transporter in roots of NM and Ri plants. Expression was studied by RT-qPCR in root tips 2 months after mycorrhization. Transcript levels were normalized to *V. vinifera ACTIN* and *EF1*α transcript levels. Fold change (2^−ΔΔCT^ method) indicates normalized expression levels in Ri roots compared to the mean of the normalized expression levels measured in NM roots. Data are the mean ± SD of 12 biological replicates. Asterisks indicate significant difference in gene expression between NM and Ri conditions (Kruskall and Wallis, *p* < 0.001).

Microscopic observations of cleared and stained roots revealed the presence of mycorrhizal structures in roots from Ri plants. In contrast, no fungal structure was found in the roots of NM plants (Figure 2A). In the colonized plants, mycorrhization frequency (F) ranged from 93 to 100%; intensity (I) ranged from 69 to 94%, and arbuscule content in colonized roots (a) ranged from 66 to 98%, showing a high level of root colonization (Figure 2B). To check the functionality of the mycorrhiza, we studied the expression of the grapevine phosphate transporter *VvPht1.2* and the *R. irregularis* hexose transporter *RiMST2*. *VvPht1.2* expression was previously reported to be highly induced, following grapevine colonization with *F. mosseae* (Valat et al., 2018). *RiMST2* is a major component for hexose uptake by the AM fungus and is essential for functional symbiosis (Helber et al., 2011). As shown in Figure 2C, an intense expression of these two marker genes was detected in mycorrhized roots. These results show that grapevine roots are efficiently colonized by *R. irregularis* and the well functioning of mycorrhiza in our system.

### Changes in Primary and Specialized Metabolites Induced by Colonization With *R. irregularis*

To determine the metabolic responses in grapevine roots and leaves upon mycorrhization with Ri, we used a non-targeted approach. Two extraction-analysis methods were performed in order to cover the maximum metabolite diversity. Raw material (leaves and roots of the same plants) was firstly extracted with a phosphate buffer and analyzed by GC-MS; the remaining pellet was subsequently extracted in MeOH and analyzed with LC-MS. Impact of mycorrhization on metabolomic response was first analyzed by PCA on GC-MS and LC-MS data. To get insight into the metabolite differences between NM and Ri samples, we further analyzed the mean relative levels of each metabolite in the different conditions. Metabolites significantly different between NM and Ri conditions were identified with a fold change of at least +/– 1.5 and a *p* value < 0.05 or 0.1 (Wilcoxon test). Metabolic changes triggered by Ri colonization in roots and leaves are detailed in the following subsections.

#### Metabolomic Responses in Roots

**Figure 3:**
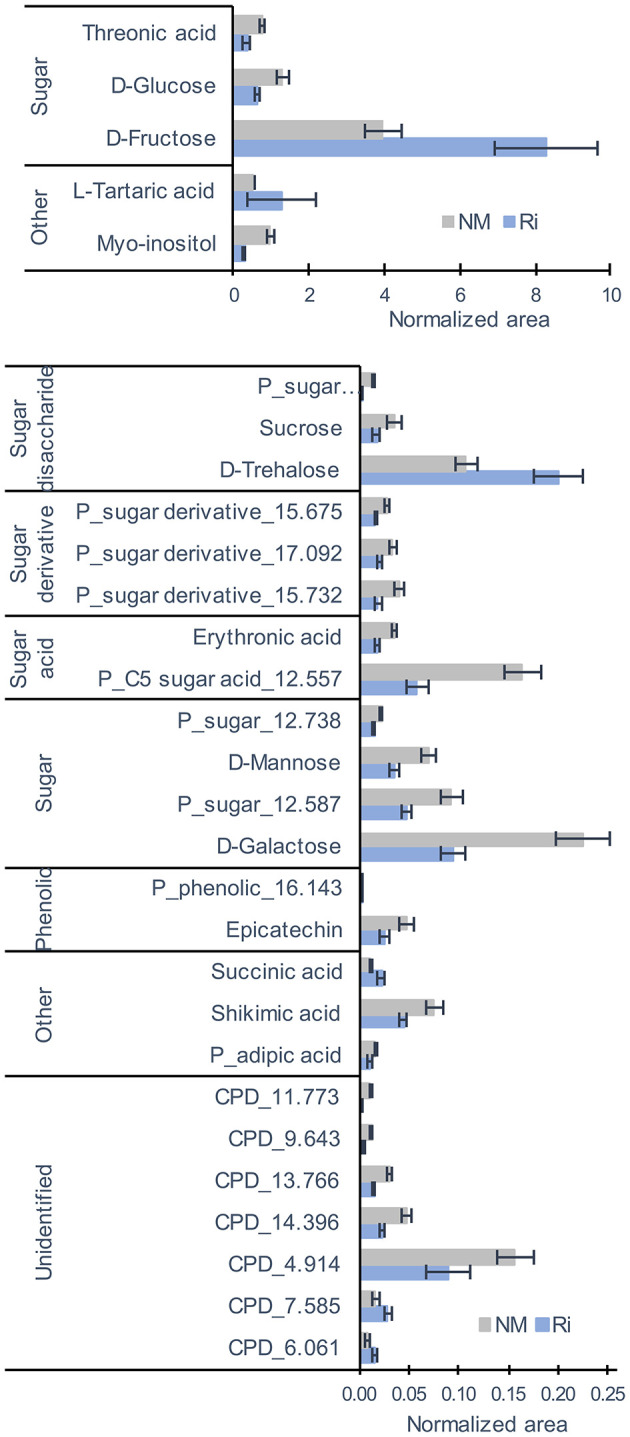
GC-MS metabolites significantly altered by the mycorrhization in roots. Contents are expressed as normalized areas for each metabolite. Data are the mean ± SE of 14 biological replicates for the NM condition and 11 biological replicates for the Ri condition. Metabolites significantly different between NM and Ri conditions were identified with a fold change of at least +/– 1.5 and a *p* value < 0.05 (Wilcoxon test).

For metabolites analyzed by GC-MS and mostly primary metabolites, a total of 83 compounds (comprising 34 identified and 23 putative compounds) were detected that belonged to sugars, sugar alcohols, sugar acids, polyols, small organic acids, amino acids, aliphatic acids, and phenolics (Supplementary Table 3). Colonization of grapevine roots with Ri resulted in significant changes in the content of primary metabolites since PCA analysis revealed a separation between NM and Ri conditions (Supplementary Figure 1). Twenty-nine metabolites were significantly impacted by the mycorrhization (Figure 3). Among identified primary metabolites, Ri colonization triggered enhanced contents of fructose, trehalose, succinic, and tartaric acids. In contrast, mycorrhizal roots had decreased levels in most of the sugar acids and sugars identified, i.e., galactose, glucose, mannose, sucrose, erythronic, and threonic acids, and also in myoinositol. A decrease in shikimic acid and epicatechin contents was also detected in mycorrhizal roots (Figure 3).

**Figure 4:**
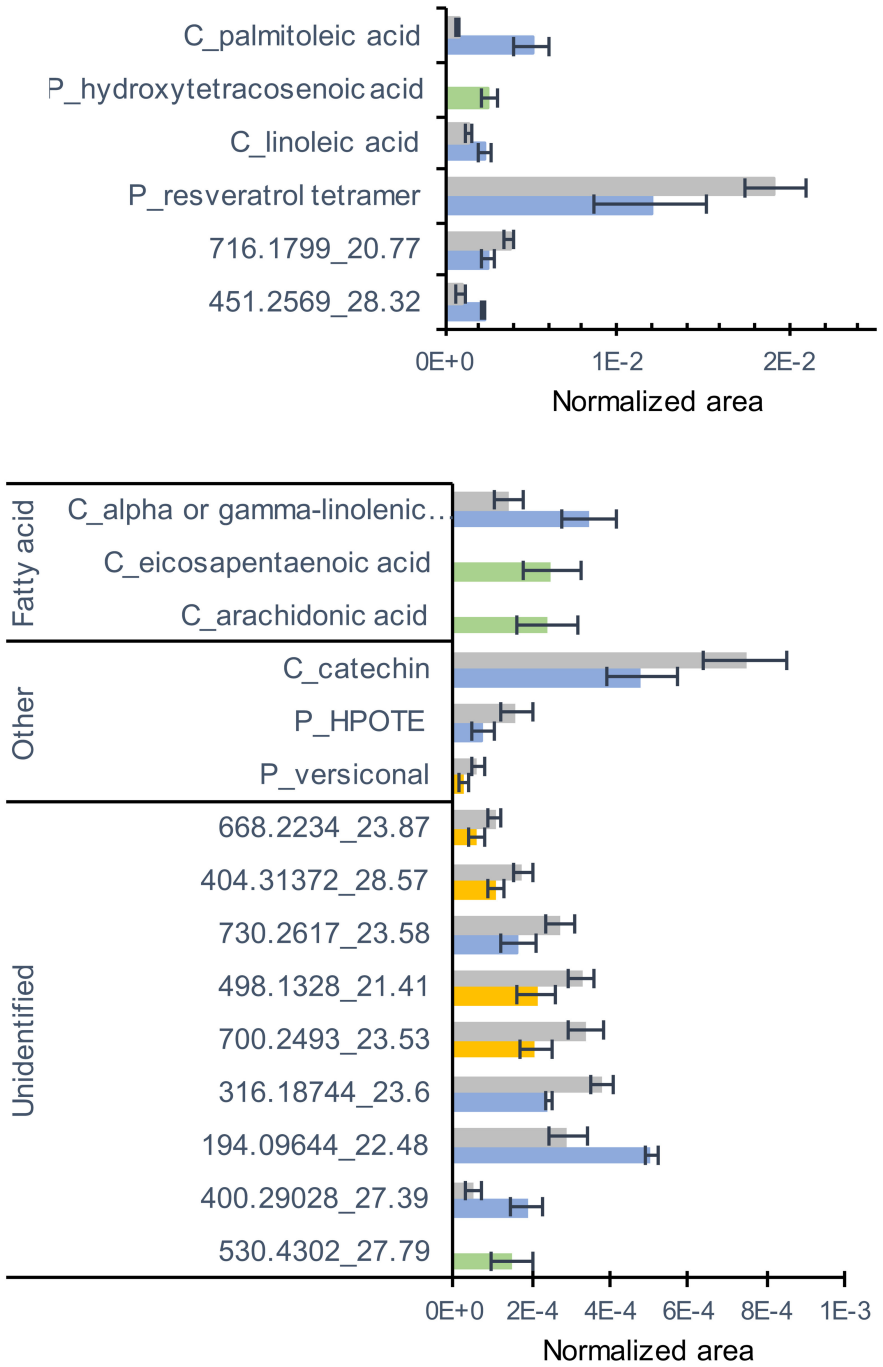
LC-MS metabolites significantly altered by the mycorrhization in roots. Contents are expressed as normalized areas for each metabolite. Data are the mean ± SE of 14 independent biological replicates for the NM condition and 11 independent biological replicates for the Ri condition. Metabolites significantly different between NM and Ri conditions were identified with a fold change of at least +/– 1.5 and a p value < 0.05 (in blue) or a *p* value < 0.1 (in yellow, Wilcoxon test). Metabolites only present in the Ri condition are shown in green.

Concerning metabolites analyzed by LC-MS, a total of 194 compounds (comprising 13 confirmed and 55 putative metabolites) were detected belonging to fatty acids, stilbenes, tannins, terpenes, flavonoids, anthraquinones, and aromatics (Supplementary Table 4). 3D-PCA of LC-MS data showed a separation between the NM and Ri (Supplementary Figure 2). Twenty-one metabolites were significantly altered by the mycorrhization (Figure 4). Interestingly, among metabolites significantly altered by mycorrhization, several fatty acids (arachidonic acid, eicosapentaenoic acid, and putative hydroxytetracosenoic acid) were solely detected in Ri root samples. Other fatty acids, i.e., C16:1 (palmitoleic acid), linoleic, and linolenic acids, were detected in NM roots but were significantly higher in Ri roots. In contrast, putative 13-HPOTE, a precursor in jasmonate biosynthesis, was less abundant in Ri samples compared to controls. Catechin content and a putative resveratrol tetramer were also lower in mycorrhizal roots (Figure 4).

**Figure 5:**
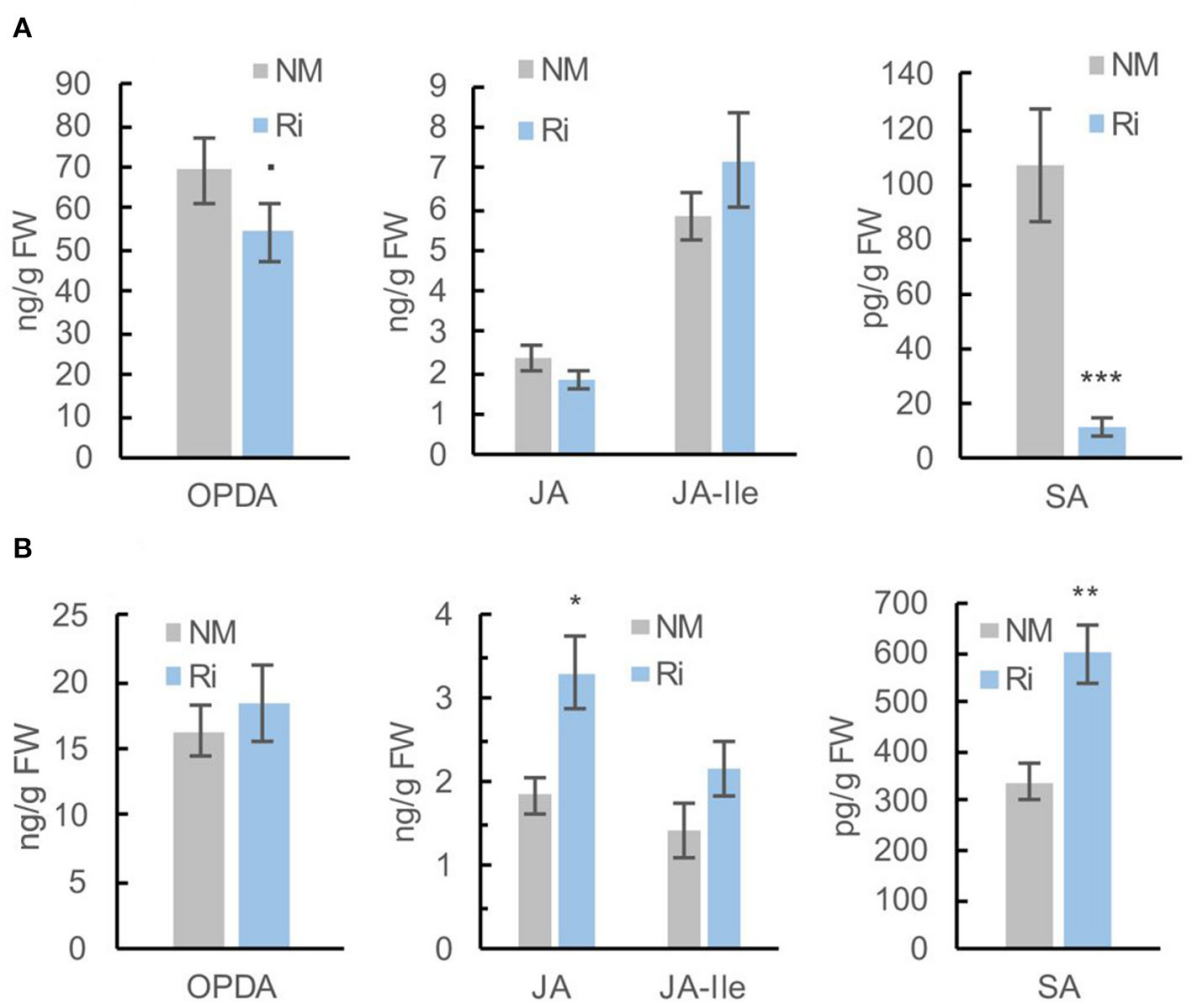
Levels of jasmonates (OPDA, JA, and JA-Ile) and salicylic acid (SA) in roots **(A)** and leaves **(B)** of Ri and NM grapevines, 2 months after mycorrhization. Data are mean ± SE of 8 independent biological replicates. Asterisks indicate significant differences between NM and Ri conditions (Kruskall and Wallis, ^***^p < 0.001, ^**^p < 0.01, ^*^p < 0.05, ^·^*p* < 0.1).

Since 13-HPOTE is a precursor of the plant hormone jasmonic acid, we further quantified jasmonates (12-oxophytodienoic acid: OPDA, jasmonic acid: JA and the jasmonoyl-isoleucine: JA-Ile) in the roots of Ri and NM plants by a targeted LC-MS method. The plant hormone salicylic acid has also been involved in the signaling of plant-AMF associations (Zamioudis and Pieterse, 2012). We thus also quantified the levels of free salicylic acid (SA), which was detected in the same extracts. The levels of OPDA were slightly lower in Ri roots compared with NM roots, while the contents in JA and JA-Ile were not significantly different. In contrast, the levels of free SA were significantly decreased in mycorrhizal roots (Figure 5A).

#### Metabolomic Responses in Leaves

**Figure 6:**
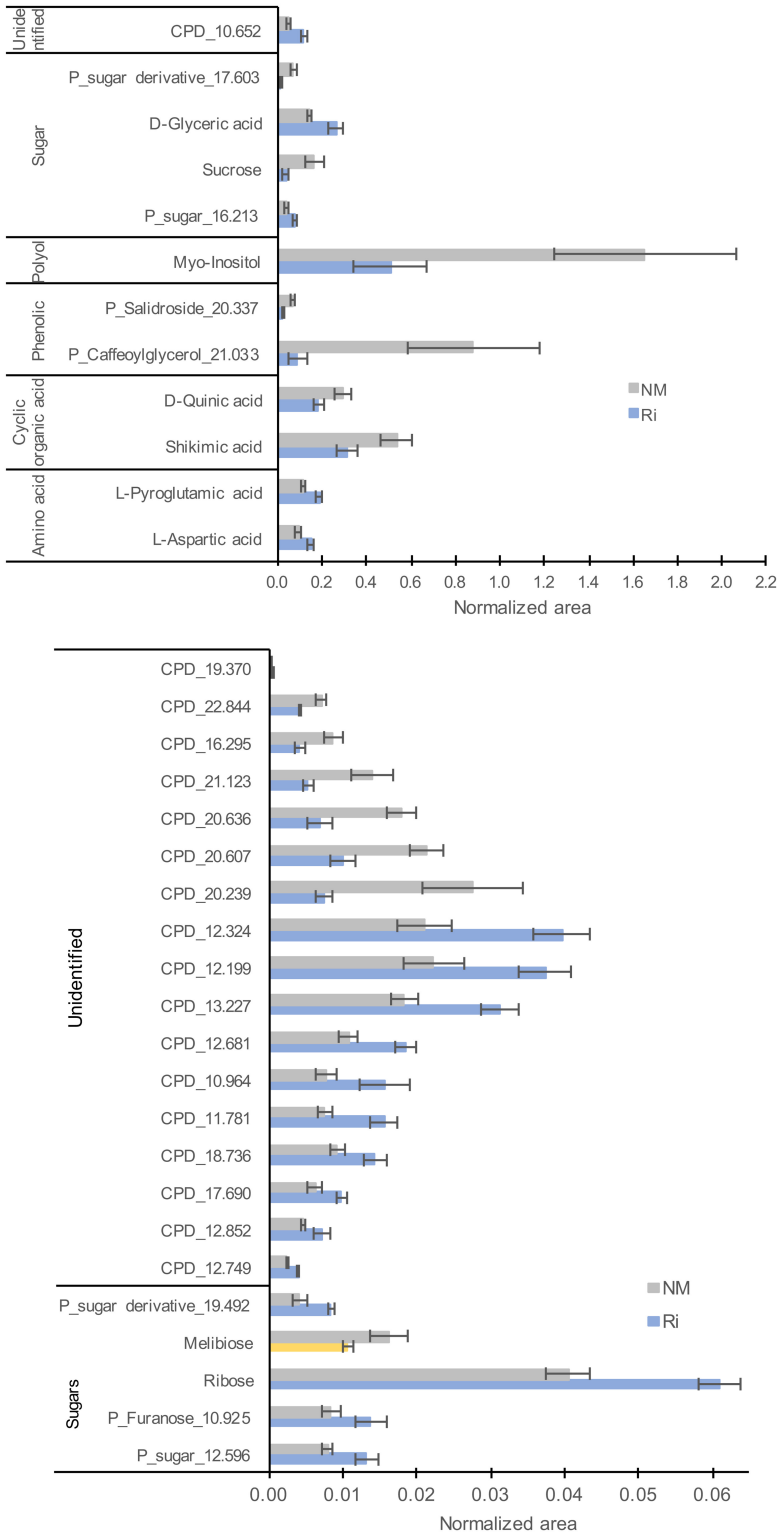
GC-MS metabolites significantly altered by the mycorrhization in leaves. Contents are expressed as normalized areas for each metabolite. Data are the mean ± SE of 14 independent biological replicates for the NM condition and 11 for the Ri condition. Metabolites significantly different between NM and Ri conditions were identified with a fold change of at least +/– 1.5 and a *p* value < 0.05 (in blue) or a *p* value < 0.1 (in yellow, Wilcoxon test).

For metabolites analyzed by GC-MS, a total of 135 compounds (including 32 confirmed and 28 putative metabolites) were detected belonging to sugars, sugar lactones, sugar alcohols, sugar acids, polyols, organic acids, amino acids, and phenolics (Supplementary Table 5). 3D-PCA revealed an impact of mycorrhization on leaf GC-MS metabolites since the NM and Ri conditions clustered in two groups (Supplementary Figure 3). Thirty-four metabolites were significantly impacted by the mycorrhization. Among identified metabolites, Ri colonization triggered decreased contents in sucrose, melibiose, myoinositol, shikimic, and quinic acids. Putative caffeoylglycerol was also one of the most downregulated compounds. In contrast, contents in ribose, pyroglutamic acid, aspartic, and glyceric acids were enhanced in mycorrhizal leaves (Figure 6).

**Figure 7:**
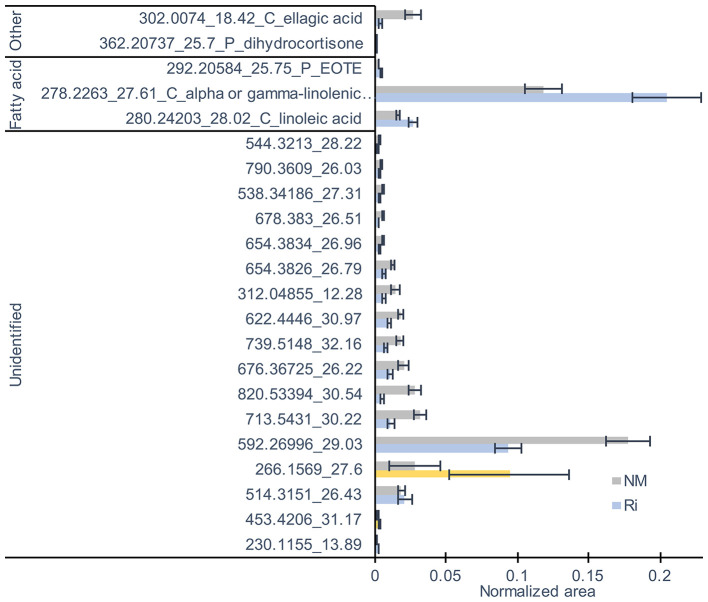
LC-MS metabolites significantly altered by the mycorrhization in leaves. Contents are expressed as normalized areas for each metabolite. Data are the mean ± SE of 14 independent biological replicates for the NM condition and 11 for the Ri condition. Metabolites significantly different between NM and Ri conditions were identified with a fold change of at least +/– 1.5 and a *p* value < 0.05 (in blue) or a *p* value < 0.1 (in yellow, Wilcoxon test).

Concerning metabolites detected by LC-MS, a total of 82 compounds (including eight confirmed and nine putative metabolites) belonging to fatty acids, steroids, tannins, and aromatics were detected (Supplementary Table 6). 3D-PCA showed that mycorrhization had no strong effect on leaf LC-MS metabolites since the two conditions were not clearly separated (Supplementary Figure 4). However, 22 metabolites were significantly impacted in the leaves by the mycorrhization. Among compounds significantly altered, mycorrhization triggered decreased contents in ellagic acid and increased contents in linoleic acid, linolenic acid, and putative EOTE, another jasmonate biosynthesis precursor (Figure 7).

Interestingly, further quantification of OPDA, JA, JA-Ile, and SA in leaves revealed that the levels of JA and SA were significantly higher in Ri leaves compared with NM leaves. Contents in OPDA and JA-Ile were not significantly different between the two conditions (Figure 5B).

### Changes in the Expression of Genes Involved in Defense Responses, JA Biosynthesis, and Sugar Transport Induced by Colonization With *R. irregularis*

To get further insight into the effect of mycorrhization on grapevine defenses and sugar metabolism, we performed a targeted transcriptomic analysis by RT-qPCR. The choice of the candidate genes was guided by the results of the metabolomic analysis, indicating modifications of fatty acids, jasmonates, and sugar metabolisms. In addition, a preliminary analysis in our laboratory of the expression of 96 defense genes with a Fluidigm 9,696 dynamic array (Dufour et al., 2016) in Ri and NM plants showed strong induction of the expression of *PR* genes of the PR6 and PR7 families in Ri roots.

In roots, we thus studied more particularly the expression of genes-encoding PR proteins (*VvPR6 bis, VvPR7*, and *VvPR7 bis*), genes involved in JA biosynthesis (*VvLOX3, VvLOX9, VvAOS1*, and *VvAOC1*), genes involved in sugar transport (*VvSWEET4, VvSWEET12, VvSUC11, VvSUC12*, and *VvSUC27*), and sugar metabolism (cytoplasmic invertase *VvCIN*, cell wall invertase *VvWINV*).

Candidate genes studied in leaves were *VvWRKY2* (defense signalization), genes involved in sugar transport and metabolism (*VvSWEET17c, VvHT1, VvHT5, VvSUC11, VvSUC12, VvSUC27, VvCIN2*, and *VvWINV*), and genes involved in JA biosynthesis (*VvLOX3, VvLOX9, VvAOS5*, and *VvAOC1*).

It should be noticed that preliminary analysis of the expression of *PR* genes that could be regulated by the SA (*PR1* and *PR2*) or JA (proteinase inhibitor *PIN* and *PR14*) defense hormones was not significantly affected by the mycorrhization in both roots and leaves (Supplementary Figure 5).

#### Transcriptomic Responses in Roots

**Figure 8:**
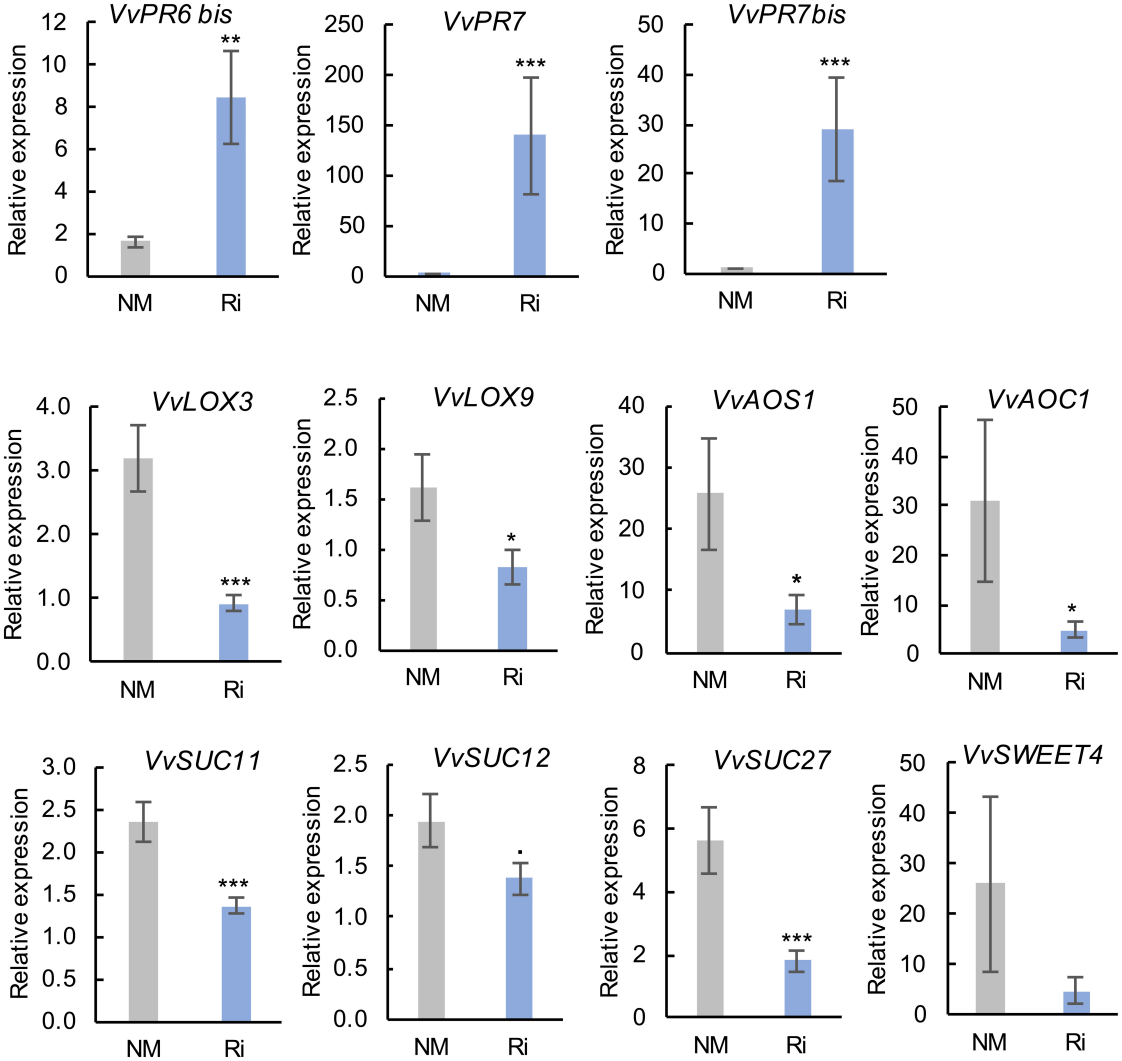
Expression of genes related to defense responses and sugar transport and metabolism in roots of NM and Ri plants. Expression was studied by RT-qPCR in root tips 2 months after mycorrhization. Transcript levels were normalized to *V. vinifera ACTIN* and *EF1*α transcript levels. For each gene, relative expression was obtained with the 2^−ΔΔCT^ method and indicates mean normalized expression in the different conditions compared with normalized expression in the plant showing the lowest expression level (highest CT value), which was set to 1. Data are the mean ± SE of 12 independent biological replicates for *VvLOX3, VvLOX9, VvAOS1, VvSUC11, VvSUC12*, and *VvSUC27*. Data are the mean ± SE of six independent biological replicates for *VvSWEET4*. Asterisks indicate significant difference in gene expression between NM and Ri conditions (Kruskall and Wallis, ^***^p < 0.001, ^**^p < 0.01, ^*^p < 0.05, ^·^*p* < 0.1).

Colonization of grapevines with Ri triggered a strong induction of *VvPR6bis, VvPR7*, and *VvPR7bis* expression in roots. On the other hand, mycorrhization resulted in significant lower expression of several genes involved in JA biosynthesis (*VvLOX3, VvLOX9, VvAOS1*, and *VvAOC1*, Figure 8). Similarly, several genes involved in sucrose transport were downregulated in Ri roots (*VvSUC11, VvSUC12*, and *VvSUC27*) (Figure 8). The expression of the hexose transporter *VvSWEET4* also seems downregulated in Ri roots, although the difference between NM and Ri roots is not significant. In contrast, expression of a SWEET transporter putatively involved in disaccharide transport (*VvSWEET12*) and of cytoplasmic and cell wall invertases (*VvCIN2* and *VvWINV*) was not significantly altered by mycorrhization (Supplementary Figure 6).

#### Transcriptomic Responses in Leaves

**Figure 9:**
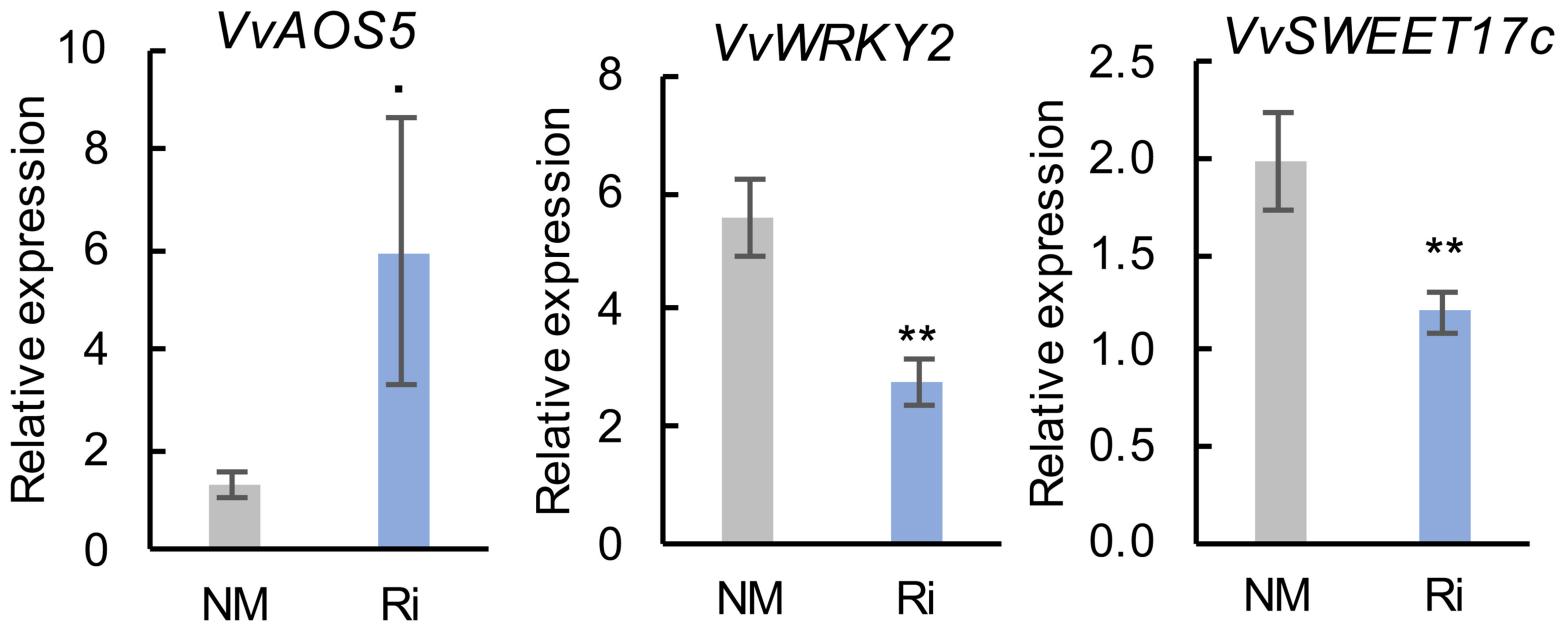
Expression of genes related to defense responses and sugar transport and metabolism in leaves of NM and Ri plants. Expression was studied by RT-qPCR 2 months after mycorrhization. Transcript levels were normalized to *V. vinifera ACTIN* and *EF1*α transcript levels. For each gene, relative expression was obtained with the 2^−ΔΔCT^ method and indicates mean normalized expression in the different conditions compared with normalized expression in the plant, showing the lowest expression level (highest CT value), which was set to 1. Data are the mean ± SE of 12 independent biological replicates for *VvAOS5, VvSWEET17c*, and *VvWRKY2*. Asterisks indicate significant difference in gene expression between NM and Ri conditions (Kruskall and Wallis, ^**^p < 0.01, ^*^p < 0.05, ^·^*p* < 0.1).

In contrast to roots, mycorrhization had less impact on the expression of genes involved in sugar transport and JA biosynthesis since there was no significant difference in the expression of most analyzed genes (Supplementary Figure 7). However, we observed some induction of the expression of *VvAOS5*. In addition, root colonization by Ri resulted in significantly lower expression of *VvWRKY2* and of the putative hexose vacuolar transporter *VvSWEET17c* (Figure 9).

## Discussion

### AMF Colonization Triggers Strong Reprogramming of Sugar Metabolism in Grapevine

In this study, we show that colonization of grapevine roots with Ri leads to major changes in primary metabolites, especially sugars and lipids. These results are in accordance with the concept of nutrient exchanges between plants and AM fungi. Indeed, it is assumed that AM fungi acquire carbon from their host plant in the form of sugars, and it has been reported more recently that lipids represent an alternative carbon source (Roth and Paszkowski, 2017).

Concerning sugar metabolism, mycorrhization with Ri led to a decrease in glucose, galactose, and sucrose, as well as an increase in fructose in roots. In leaves, we observed a significant decrease in sucrose and myoinositol. A decrease in hexoses in grapevine roots likely results from their transfer to the AMF. Indeed, it is known that AM fungi acquire sugars from host plants in the form of hexoses, glucose being the predominant form imported in the intra-radical mycelium (Roth and Paszkowski, 2017; Kaur and Suseela, 2020). In contrast to glucose, higher fructose levels were observed in mycorrhizal roots and may originate from sucrose cleavage by invertases, followed by preferential glucose uptake by the AMF. At the beginning of colonization with the AM fungus, several studies have reported higher hexose concentration in the roots of diverse plants, probably resulting from enhanced sucrose cleavage (Kaur and Suseela, 2020). At later stages (similar to our study, 2 months after mycorrhization), lower sugar concentrations were detected in mycorrhized compared with non-mycorrhized roots in soybean (Schubert et al., 2004), as a consequence of further hexose metabolization by mycorrhizal sink roots. Regarding sugar contents in leaves, it is well assumed that root colonization with AM fungi enhances the source to sink flux, resulting in sucrose redirection from leaves to roots (Roth and Paszkowski, 2017). Accordingly, we observed a decrease in sucrose in grapevine leaves following mycorrhization, suggesting a higher sucrose export from source leaves. In *Medicago truncatula* (*M. truncatula*), a decrease in total soluble sugars was also reported in leaves after mycorrhization with Ri (Doidy et al., 2012a). In plants, long-distance sugar transport is mediated by SUT transporters that are involved in cellular proton-coupled sucrose uptake. In source leaves, SUT are involved in sucrose loading into the phloem for long-distance transport, whereas they function in sucrose uptake in cells of sink tissues, such as roots (Doidy et al., 2012b). Unexpectedly, following grapevine inoculation with Ri, we observed downregulation of expression of all SUT transporters analyzed in roots, as well as downregulation of *VvSUT27* and no difference in expression of *VvSUT11*/*12* in leaves. In contrast, in the *M. truncatula*/*R. irregularis* symbiosis, Doidy et al. (2012a) reported overall upregulation of almost all SUT transporters in leaves and roots, following colonization with the AMF. Similarly, in tomato, following inoculation with *G. mosseae*, the expression of all SUT transporters was induced in leaves, whereas only *SlSUT1* and *SlSUT4* were upregulated in roots (Boldt et al., 2011). It is possible that phloem loading in grapevines, as in other woody species, involves passive transport through a symplastic pathway independent of SUT transporters (Turgeon, 2010). Overall, SUT transporters could have opposite roles in plant-AMF symbiosis since a knockdown of the tomato SUT transporter *SlSUT2* resulted in increased mycorrhization (Bitterlich et al., 2014), whereas potato plants overexpressing *SUT1* exhibited increased mycorrhization under high phosphorus availability (Gabriel-Neumann et al., 2011). In grapevines, VvSUC11, 12, and 27 were characterized as plasma membrane sucrose H^+^ symporters involved in sucrose uptake (Cai et al., 2019). If these transporters facilitate the loading of sucrose from the periarbuscular matrix into root cortical cells, their downregulation could favor sugar transfer toward the AMF. Concerning SWEET transporters, no significant difference was found between Ri and NM plants in the expression of *VvSWEET4* and *VvSWEET12* in roots. In leaves, the expression of *VvSWEET17c*, a putative tonoplast hexose transporter, was lower in mycorrhizal plants, whereas the expression of the hexose transporter *VvHT5* was not modified. In contrast to our results, Manck-Götzenberger and Requena (2016) reported major transcriptional changes of *SWEETs* in potato roots induced by the AM fungus *R. irregularis*. In addition to sugar transporters, extracellular invertases would play a role in supplying the AM fungus with hexoses (Schaarschmidt et al., 2006). However, no difference in the expression of both cytoplasmic invertase or cell wall invertase was found between mycorrhizal and non-mycorrhizal roots. It is possible that basal plant invertase activity is sufficient for apoplastic sucrose hydrolysis in Ri-colonized roots.

Our results thus show a different regulation pattern for SUTs and SWEETs transporters in grapevines compared with other plants and suggest a different regulation of carbon partitioning in grapevines compared with herbaceous species. Although we observed overall downregulation of sugar contents in both roots and leaves of Ri grapevines, it has to be noticed that it did not lead to a negative impact on grapevine growth.

### AMF Colonization Triggers Strong Reprogramming of Fatty Acid Metabolism in Grapevine

Other primary metabolites strongly impacted in mycorrhizal roots belong to the lipid family, in agreement with the fact that lipids constitute a main carbon store in AMF (Keymer et al., 2017). It has been recently demonstrated that, in addition to sugars, lipids are also major nutrients synthesized by plants and transferred to AMFs, which are fatty acid auxotrophs (Jiang et al., 2017; Luginbuehl et al., 2017). In addition, lipids are essential for the establishment of symbiosis, either as signals or as constituents of the periarbuscular membrane (Vijayakumar et al., 2016). Upon arbuscule formation, a specific lipid biosynthesis pathway is induced in plant cells to synthesize high levels of palmitic acid (C16:0), a precursor of 16:0 β-monoacylglycerol, which can be exported to the symbiont across the periarbuscular membrane (Wipf et al., 2019). Subsequently, AMF are able to elongate and desaturate fatty acids provided by the plant (Trépanier et al., 2005). In this study, higher contents in palmitoleic acid (C16:1) were found in Ri roots, and several C20–24 fatty acids (arachidonic acid, eicosapentaenoic acid, and putative hydroxytetracosenoic acid) were only detected in mycorrhized roots. C16:1 fatty acids, which are monounsaturated fatty acids produced by desaturation of palmitic acid have been already described as good indicators of fungal AM development (Van Aarle and Olsson, 2003; Trépanier et al., 2005). C20 fatty acids are likely produced by the AMF from fatty acids provided by the plant. Indeed, in the *Lotus japonicus*/*R. irregularis* symbiosis, arachidonic acid (C20:4) and eicosapentaenoic acid (C20:5) were shown to be synthesized by the symbiont (Vijayakumar et al., 2016).

Furthermore, we show that AMF colonization results in significantly higher contents in linoleic (C18:2) and linolenic (C18:3) acids in roots and in higher levels in putative EOTE, linoleic, and linolenic acids in leaves. Besides their role as a carbon source for the AMF, fatty acids, and especially unsaturated fatty acids, play a key role in plant defense (Lim et al., 2017). Interestingly, Xing and Chin (2000) have shown that eggplants with enhanced levels of palmitoleic acid (C16:1) and C16:3 due to overexpression of a yeast Δ9 desaturase have better resistance to *Verticillium dahliae*. In addition, increased levels of C18:2 and C18:3 lead to better resistance to *Colletotrichum gloeosporioides* in avocado (Wang et al., 2004) and *Pseudomonas syringae* in tomato (Yaeno et al., 2004). In another study in bean, Ongena et al. (2004) reported that the accumulation of C18:2 and C18:3 fatty acids is associated with enhanced resistance to *B. cinerea* triggered by rhizobacteria (Ongena et al., 2004).

### AMF Colonization Results in Contrasting Effect on JA and SA Metabolism in Roots and Leaves of Grapevine

Whereas the levels of several fatty acids involved in JA biosynthesis (linoleic and mostly linolenic acid) are enhanced in Ri roots, mycorrhization results in some extent in downregulation of the JA pathway. Indeed, the expression of several enzymes involved in JA biosynthesis (*VvLOX3, VvLOX9, VvAOS1*, and *VvAOC1*) was lower in Ri roots, and a lower content in OPDA was detected. It is known that linoleic and linolenic acids can be synthesized by the plant or the AMF in roots and could be precursors for longer chain fatty acids (C20) produced by the fungus (Vijayakumar et al., 2016). It is possible that the α-linolenic acid pool is directed toward the synthesis of AMF specific C20 fatty acids (eicosapentaenoic acid) and less available for the JA biosynthesis in roots. However, contents in JA and in the active form JA-Ile were not significantly reduced by the mycorrhization. It is important to notice that transcriptomic and metabolomic analyses were both performed 2 months post-mycorrhization, and it is possible that the effect of JA biosynthesis gene downregulation on metabolite content is visible at later time points. Our results contrast with other studies reporting that roots of AM plants contain high levels of jasmonates (Wasternack and Hause, 2013). In addition, induction of JA biosynthesis has been demonstrated in mycorrhizal roots of barley (Hause, 2002). Although a positive role for jasmonates in mycorrhizal colonization has been suggested in several studies, contradictory results exist on this subject (Foo et al., 2013).

In addition, a significant reduction in free SA levels was evidenced in Ri roots. It is possible that the downregulation of hormonal pathways involved in defense response signaling allows better colonization of roots by the AMF. Indeed, a negative effect of SA signaling on root colonization by AMF has been shown, and it is known that SA-mediated defense responses are suppressed by mycorrhizal fungi (Zamioudis and Pieterse, 2012).

Importantly, an opposite effect of mycorrhization on defense hormones was evidenced in leaves. Indeed, leaves of Ri plants were characterized by higher JA and SA levels compared with NM plants. JA-Ile levels were also increased to some extent in Ri plants, although not significantly. Higher JA levels in Ri leaves are consistent with increased levels of linolenic acid, a precursor of this hormone, as well as with the higher expression of *VvAOS5* that could be involved in the synthesis of the JA precursor OPDA. In another study, Pedranzani et al. (2016) also reported a significant increase in the levels of 12-OPDA, JA, and 12-OHJA in shoots of *Digitaria eriantha* after colonization with Ri. In the same way, AMF-colonized tomato plants had higher lipoxygenase activity and methyljasmonate levels that could be involved in the onset of MIR against the vascular pathogen *Fusarium oxysporum* and the leaf pathogen *Alternaria alternata* (Nair et al., 2015a,b). It is possible that enhanced defense hormone levels in leaves could induce a primed state that enables grapevine to better respond to foliar pathogens.

### AMF Colonization Triggers Less Alterations in Specialized Metabolites

Few studies have reported the effect of AMF colonization alone on leaf specialized metabolites in grapevine. However, Torres et al. (2018b) showed higher flavonol levels in leaves of Tempranillo after inoculation of fruit-bearing cuttings with a mixture of five AMF. In another study, Eftekhari et al. (2012) found high levels of quercetin in hardwood cuttings of two Iranian varieties after inoculation with *G. mosseae*.

In this study, whereas AMF colonization had strong impact on primary metabolism, it had less direct effect on specialized metabolism. Indeed, even if a number of stilbene derivatives were detected in roots (Supplementary Table 4), their content was not significantly altered by the mycorrhization. In leaves, fewer specialized metabolites were detected, and ACP analysis of LC-MS metabolites did not show a clear separation between the two conditions. The metabolites and metabolic pathways impacted by the AMF colonization are summarized in Supplementary Figure 8. However, we observed decreased contents in catechin and epicatechin (flavanols) in roots as well as decreased levels in shikimic acid (a precursor of aromatic amino acids used in specialized metabolite synthesis), quinic acid (organic acid), and ellagic acid in leaves. Ellagic acid is the precursor of hydrolyzable tannins found in grapevine leaves and canes (Goufo et al., 2020). One explanation could be that there is a competition between primary and specialized metabolite synthesis and that sugars directed to mycorrhized sink roots or used for fatty acid synthesis are less available for specialized metabolite synthesis. To support this hypothesis, we observed a lower sucrose content in leaves concomitant to lower levels in shikimic and ellagic acids. Mycorrhizal roots showed significant lower accumulation of a resveratrol tetramer, and it is possible that lower contents in antifungal stilbene in Ri roots favor better colonization by the AMF. No stilbene accumulation was found in leaves of mycorrhized grapevines. In another study, Bruisson et al. (2016) reported higher stimulation of stilbene biosynthesis genes and accumulation of bioactive stilbenes in leaves of mycorrhized grapevines, but only after inoculation with different types of pathogens.

### Expression of Several Defense Genes Is Strongly Modulated by AMF Colonization in Grapevine, Especially in Roots

Interestingly, colonization of grapevine roots with Ri triggered a high induction of the expression of *VvPR6 bis, VvPR7*, and *VvPR7 bis* in roots. PR proteins are inducible defense proteins expressed after plant infection with pathogens, such as oomycetes, fungi, bacteria, and viruses (Sels et al., 2008). PR6 bis is a proteinase inhibitor and belongs to a subclass of serine proteinase inhibitors that could play a role in plant defense by interacting with proteinases from bioagressors (Sels et al., 2008).

PR7 and 7 bis are plant proteases belonging to the subtilisin-like serine protease (subtilase) family of proteins involved in both mutualistic symbiosis and plant/pathogen interactions (Takeda et al., 2007; Figueiredo et al., 2016). Induction of subtilase genes was evidenced by transcriptome analysis during AM symbiosis as well as during root nodule symbiosis in different plant species (Takeda et al., 2007). Interestingly, it has been shown in *Lotus japonicus* that apoplastic subtilases support the development of arbuscular mycorrhiza. Indeed, inhibition of the subtilases SbtM1 or SbtM3 by RNAi resulted in lower intra-radical hyphae and arbuscule development (Takeda et al., 2009). In grapevine, it was previously reported that subtilisin-like proteases are induced in rootstocks colonized by *R. irregularis* or *F. mosseae* (Cangahuala-Inocente et al., 2011; Balestrini et al., 2017). In addition, subtilases may also be linked to immune priming in plants (Figueiredo et al., 2014). Indeed, several subtilases were early induced in grapevine leaves following inoculation with *P. viticola*, especially in resistant genotypes (Figueiredo et al., 2016), as well as in *Vitis pseudoreticulata* inoculated with *Erysiphe necator* (Weng et al., 2014). In this study, it is likely that grapevine subtilisin-like PR7 and PR7 bis are involved in the development of arbuscular mycorrhiza, but high levels of PR expression could also confer better resistance to root pathogens. It should be noticed that since SA levels are downregulated in Ri roots, *VvPR6* and *VvPR7* expression is likely regulated *via* SA-independent pathways. In *Lotus japonicus*, it has been reported that inhibition of GA biosynthesis or signaling repressed the AM-induced subtilisin-like SbtM1 (Takeda et al., 2015).

In leaves, mycorrhization alone had no strong effect on the expression of defense genes. However, we found downregulation of *WRKY2* expression in mycorrhized grapevine leaves. The grapevine transcription factor WRKY2 influences the lignin pathway and xylem development when expressed in tobacco, and transgenic tobacco overexpressing VvWRKY2 exhibited reduced susceptibility to several necrotrophic pathogens (Mzid et al., 2007; Guillaumie et al., 2010). However, several WRKY also function as negative regulators of plant immunity (Jiang et al., 2019). More experiments are needed to precise the role of this transcription factor in the grapevine response to AMF.

## Conclusion

In conclusion, our study provides a picture of transcriptomic and metabolomic changes induced by AMF colonization in both roots and leaves of grapevine. As we are aware that this study has been realized in controlled conditions on non-grafted grapevine, it will be interesting to complete these results by further studies closer to field conditions. However, our results reveal that enhanced levels of several unsaturated fatty acids in grapevine roots and leaves, together with higher levels of SA and JA in leaves and PR protein accumulation in roots, have the potential to confer better resistance to various pathogens in mycorrhized plants, either by direct or priming effects. Future work will also focus on the effect of mycorrhization on grapevine tolerance to bioagressors, especially leaf pathogens.

## Data Availability Statement

The original contributions presented in the study are included in the article/Supplementary Material, further inquiries can be directed to the corresponding author/s.

## Author Contributions

JC, LB, and M-LG: conceived and designed experiments. IM, HL, JC, LB, LD-B, LR, LV, and M-LG: performed experiments. IM, JC, LR, LB, and M-LG: analyzed experiments. M-LG and CB: contributed materials/analysis tools. JC and M-LG: wrote the paper. CB and JC: funding acquisition. All authors contributed to the article and approved the submitted version.

## Conflict of Interest

The authors declare that the research was conducted in the absence of any commercial or financial relationships that could be construed as a potential conflict of interest.

## Publisher's Note

All claims expressed in this article are solely those of the authors and do not necessarily represent those of their affiliated organizations, or those of the publisher, the editors and the reviewers. Any product that may be evaluated in this article, or claim that may be made by its manufacturer, is not guaranteed or endorsed by the publisher.

## Supplementary Material

Supplementary Figures 1–4 Three-dimensional principal component analysis (3D PCA) of GC-MS and LC-MS metabolite levels in roots and leaves.

Supplementary Figure 5 Expression of *PR* genes putatively regulated by SA and JA in leaves and roots of NM and Ri plants.

Supplementary Figure 6 Expression of genes related to sugar transport and metabolism in roots of NM and Ri plants.

Supplementary Figure 7 Expression of genes related to defense responses and sugar transport and metabolism in leaves of NM and Ri plants.

Supplementary Figure 8 Summary of the root and leaf metabolites in the metabolic pathways significantly affected by the mycorrhization.

Supplementary Table 1 Primers used in this study.

Supplementary Table 2 Jasmonates quantification. Target monitored ions, chromatographic retention times (RT), assigned internal standards, quantifier, and qualifier ions.

Supplementary Tables 3–6 A list of all GC-MS and LC-MS metabolites identified in roots and leaves.

Supplementary Tables 7 and 8 Melt curves of qPCR amplicons in roots and leaves.
