# Supplementary material for: Arbuscular Mycorrhizal Symbiosis Triggers Major Changes in Primary Metabolism Together With Modification of Defense Responses and Signaling in Both Roots and Leaves of Vitis vinifera
Source: Front Plant Sci. 2021 Aug 25;12:721614. doi: 10.3389/fpls.2021.721614 (PMC8424087; doi:10.3389/fpls.2021.721614)
Supplement: Supplementary Figures 1–4 — Three-dimensional principal component analysis (3D PCA) of GC-MS and LC-MS metabolite levels in roots and leaves. [file Data_Sheet_1.zip › Supplementary Figures S1-S4.DOCX]

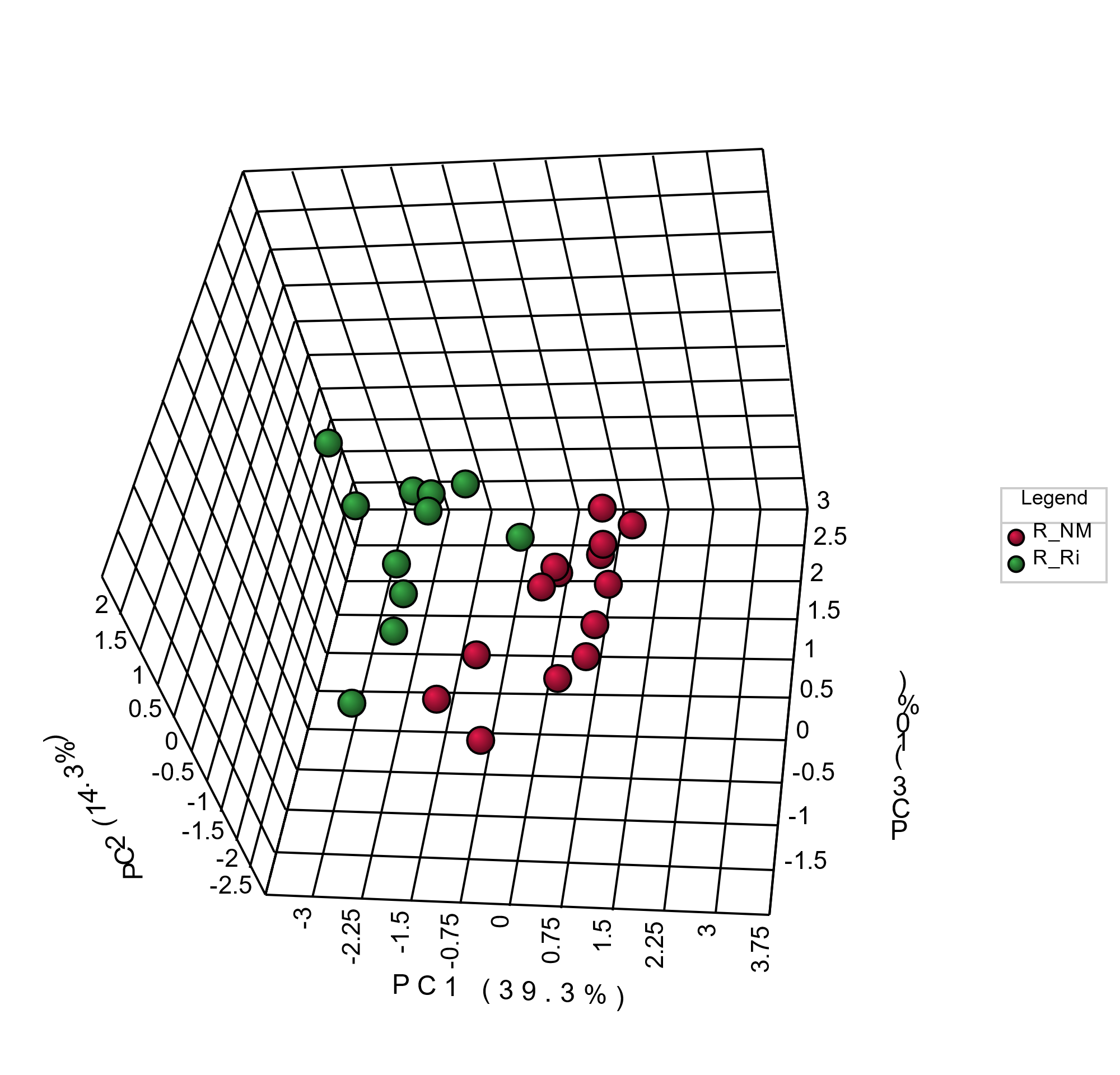

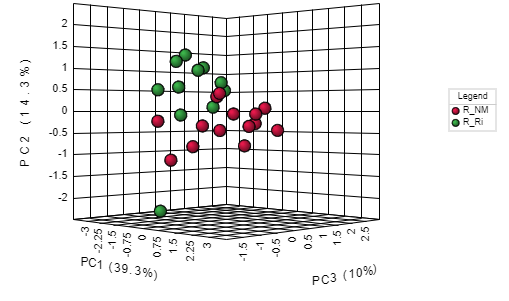


**Supplementary Figure S1: Three-Dimensional Principal Component Analysis (3D PCA) of GC-MS metabolite levels in roots.** 14 independent biological replicates are shown for non-mycorrhized condition (NM, in red) and 11 independent replicates for mycorrhized condition (Ri, in green). The three major principal components explained 64% of the cumulative variance.


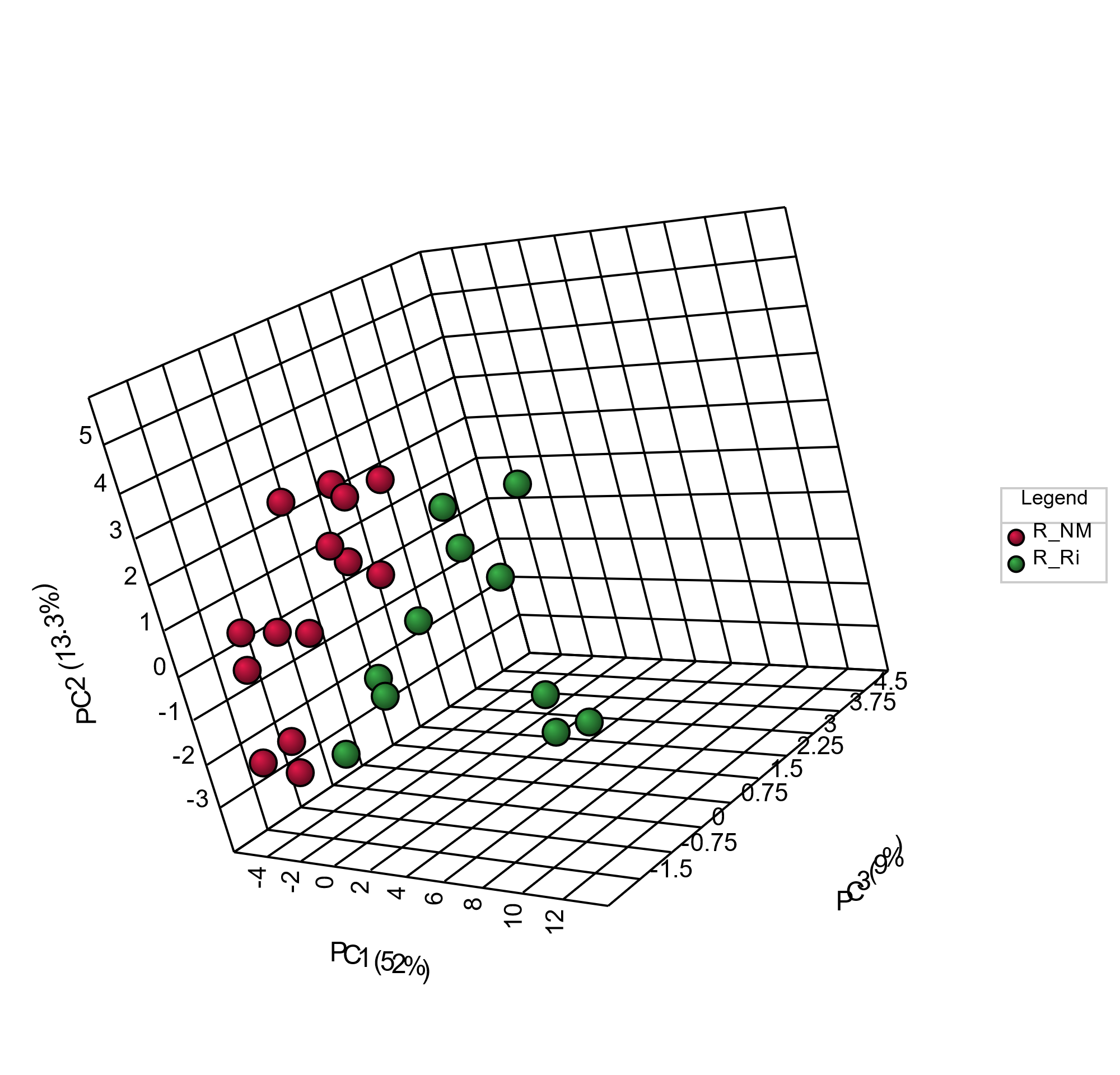

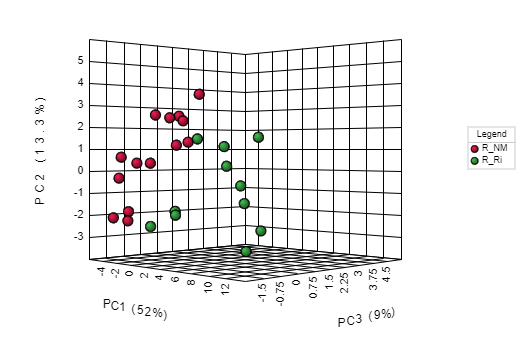


**Supplementary Figure S2: Three-Dimensional Principal Component Analysis (3D PCA) of LC-MS metabolite levels in roots.** 14 biological replicates are shown for non-mycorrhized condition (NM, in red) and 11 biological replicates for the mycorrhized condition (Ri, in green). The three major principal components explained 74% of the cumulative variance.


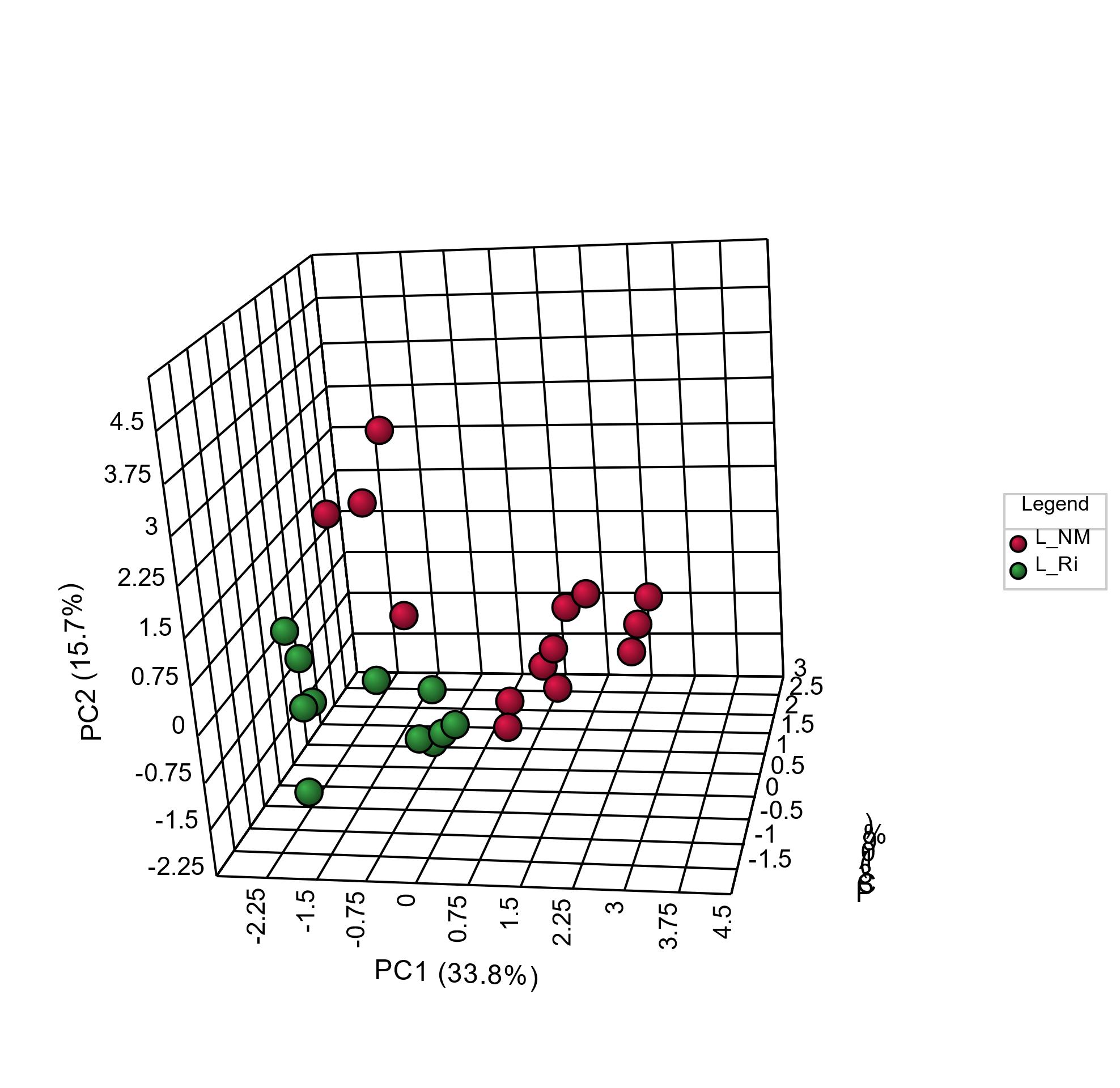

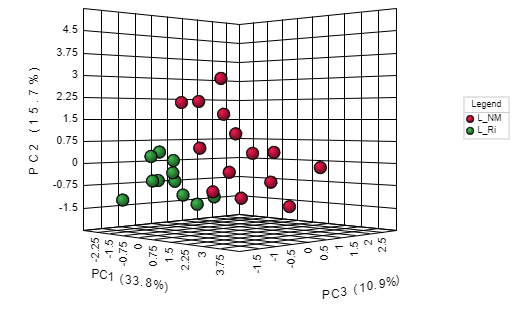


**Supplementary Figure S3: Three-Dimensional Principal Component Analysis (3D PCA) of GC-MS metabolite levels in leaves.** 14 independent biological replicates are shown for the non-mycorrhized condition (NM, in red) and 11 for the mycorrhized condition (Ri, in green). The three major principal components explained 60% of the cumulative variance.


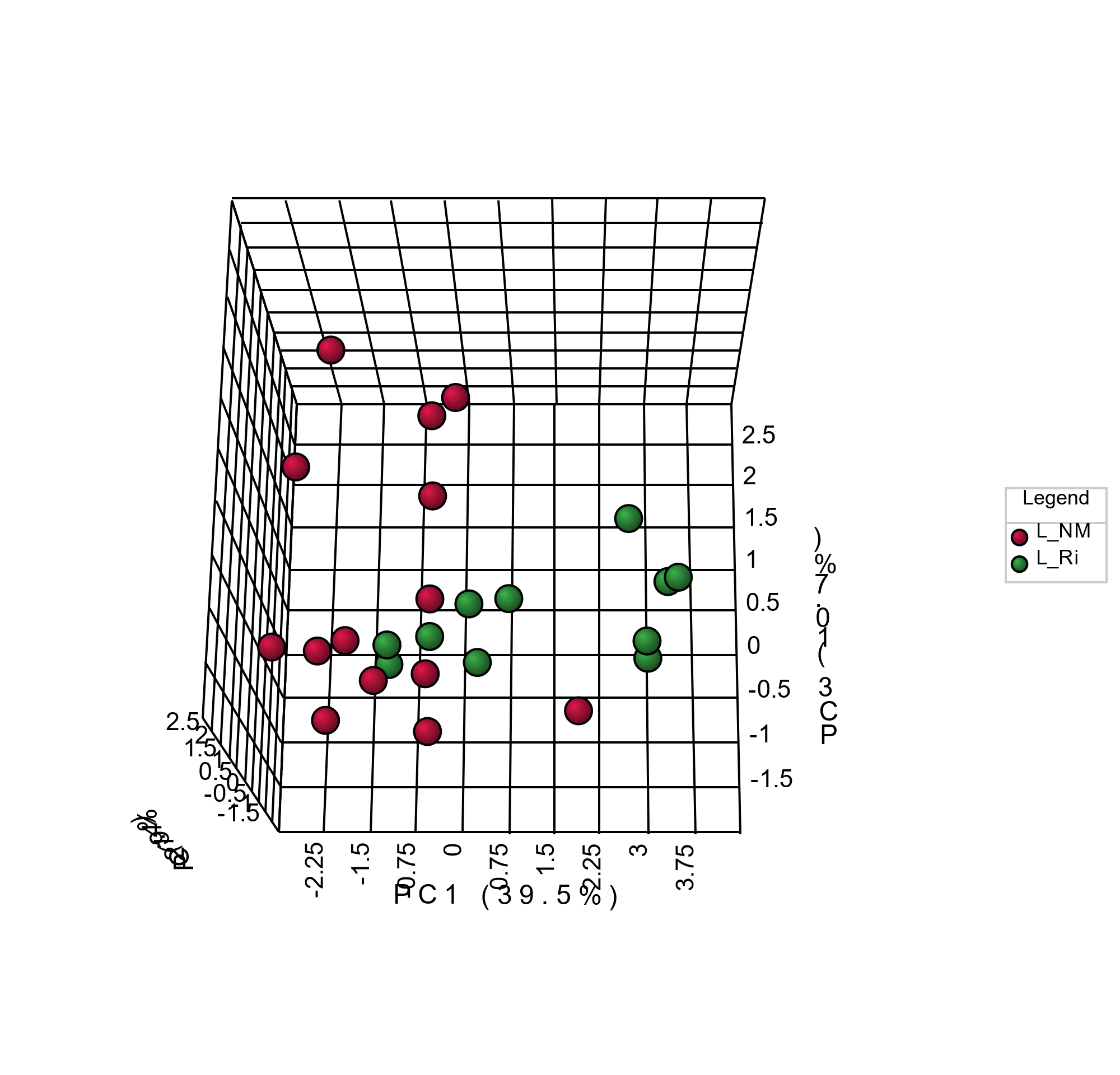

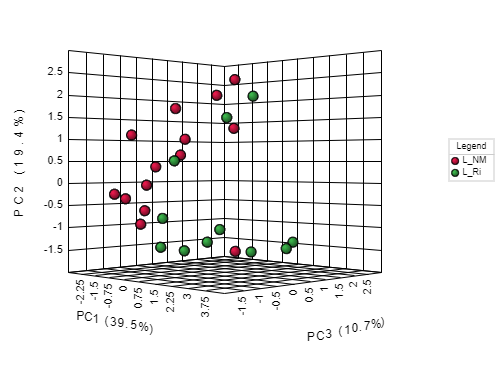


**Supplementary Figure S4: Three-Dimensional Principal Component Analysis (3D PCA) of LC-MS metabolite levels in leaves.** 14 independent biological replicates are shown for the non-mycorrhized condition (NM, in red) and 11 for the mycorrhized condition (Ri, in green). The two major principal components explained 70% of the cumulative variance.

.
