# Supplementary material for: Arbuscular Mycorrhizal Symbiosis Triggers Major Changes in Primary Metabolism Together With Modification of Defense Responses and Signaling in Both Roots and Leaves of Vitis vinifera
Source: Front Plant Sci. 2021 Aug 25;12:721614. doi: 10.3389/fpls.2021.721614 (PMC8424087; doi:10.3389/fpls.2021.721614)
Supplement: Supplementary Figures 1–4 — Three-dimensional principal component analysis (3D PCA) of GC-MS and LC-MS metabolite levels in roots and leaves. [file Data_Sheet_1.zip › Supplementary Figures S5-S8.DOCX]

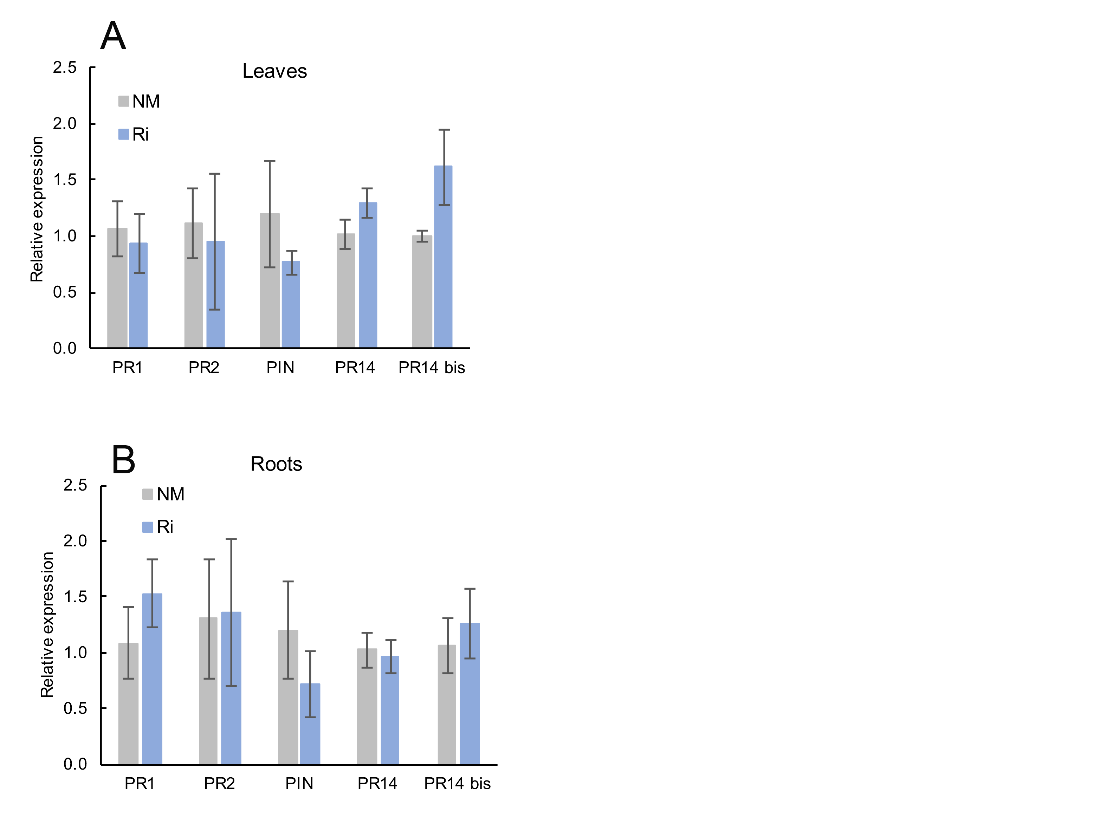


**Supplementary Figure S5.** **Expression of *PR* genes putatively regulated by SA and JA in leaves and roots of NM and Ri plants.**

Expression was studied with the NeoViGen96 chip (Dufour et al., 2016) in root tips and leaves 2 months after mycorrhization. For each gene, relative expression was calculated with the 2^-ΔΔCT^ method and indicates mean normalized expression in Ri condition compared with normalized expression in NM condition. Data are the mean ± SE of 3 independent biological replicates. *PIN*: proteinase inhibitor.


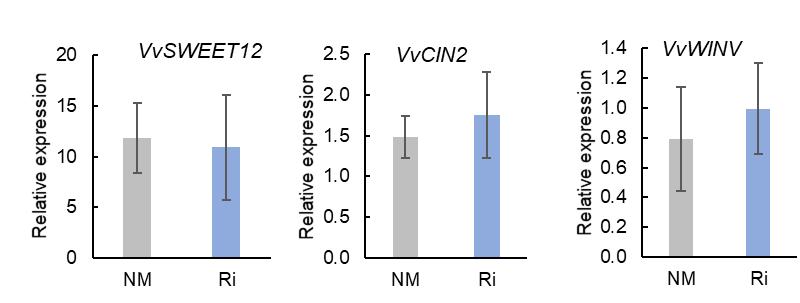


**Supplementary Figure S6. Expression of genes related to sugar transport and metabolism in roots of NM and Ri plants.**

Expression was studied by RT-qPCR in root tips 2 months after mycorrhization. Transcript levels were normalized to *V. vinifera* *ACTIN* and *EF1α* transcript levels. For each gene, relative expression was obtained with the 2^-ΔΔCT^ method and indicates mean normalized expression in the different conditions compared with normalized expression in the plant showing the lowest expression level (highest CT value), which was set to 1. Data are the mean ± SE of 12 independent biological replicates for *VvCIN2*. Data are the mean ± SE of 6 independent biological replicates for *VvSWEET12* and *VvWINV.*


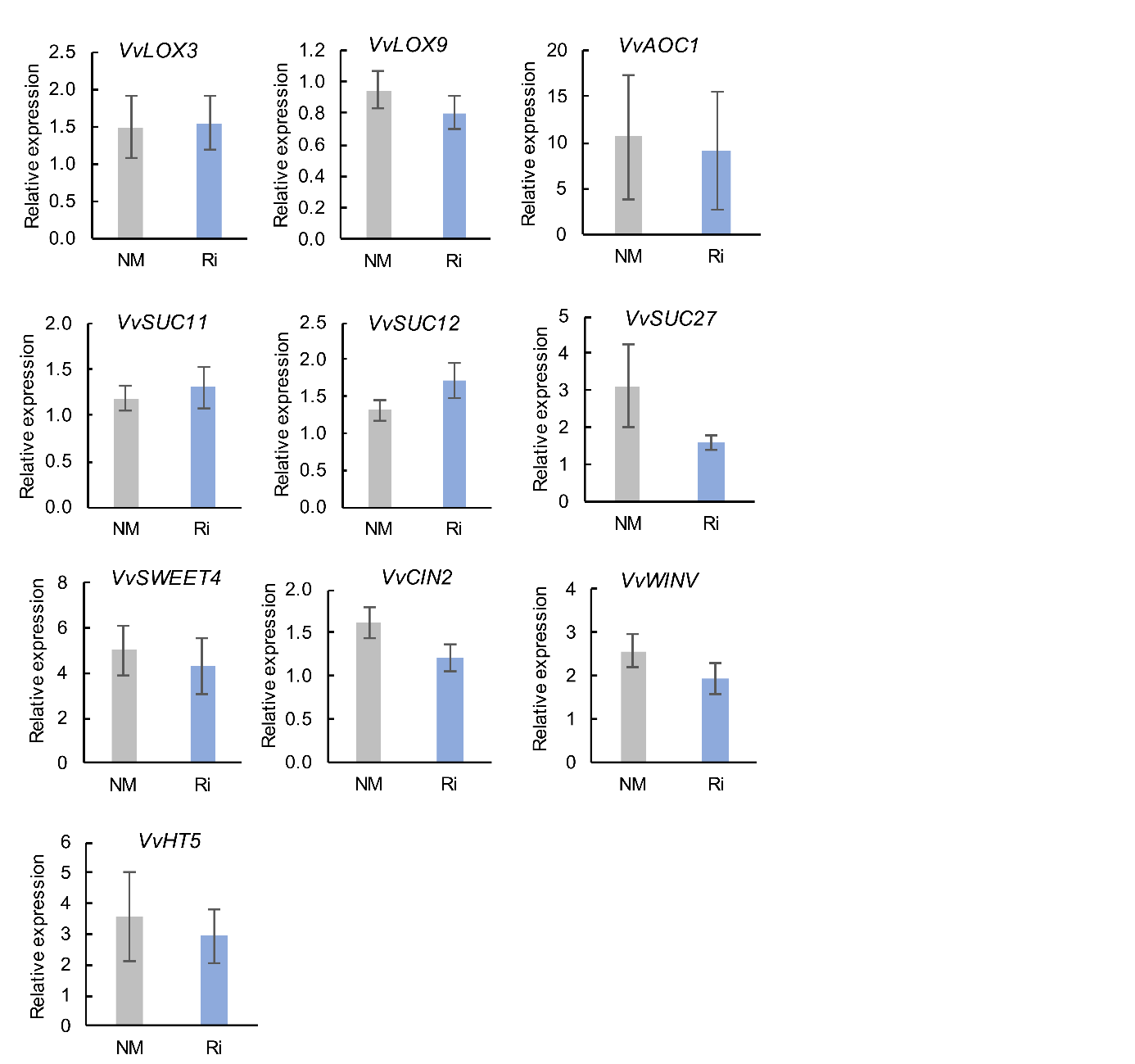


**Supplementary Figure S7. Expression of genes related to defense responses and sugar transport and metabolism in leaves of NM and Ri plants.**

Expression was studied by RT-qPCR 2 months after mycorrhization. Transcript levels were normalized to *V. vinifera* *ACTIN* and *EF1α* transcript levels. For each gene, relative expression was obtained with the 2^-ΔΔCT^ method and indicates mean normalized expression in the different conditions compared with normalized expression in the plant showing the lowest expression level (highest CT value), which was set to 1. Data are the mean ± SE of 12 independent biological replicates for *VvLOX9, VvAOC1, VvSUC27, VvSWEET4* and *VvCIN2*. Data are the mean ± SE of 6 independent biological replicates for *VvLOX3, VvSUC11, VvSUC12, VvHT5 and VvWINV.*

**Supplementary Figure S8. Summary of the root and leaf metabolites in the metabolic pathways significantly affected by the mycorrhization.**

**
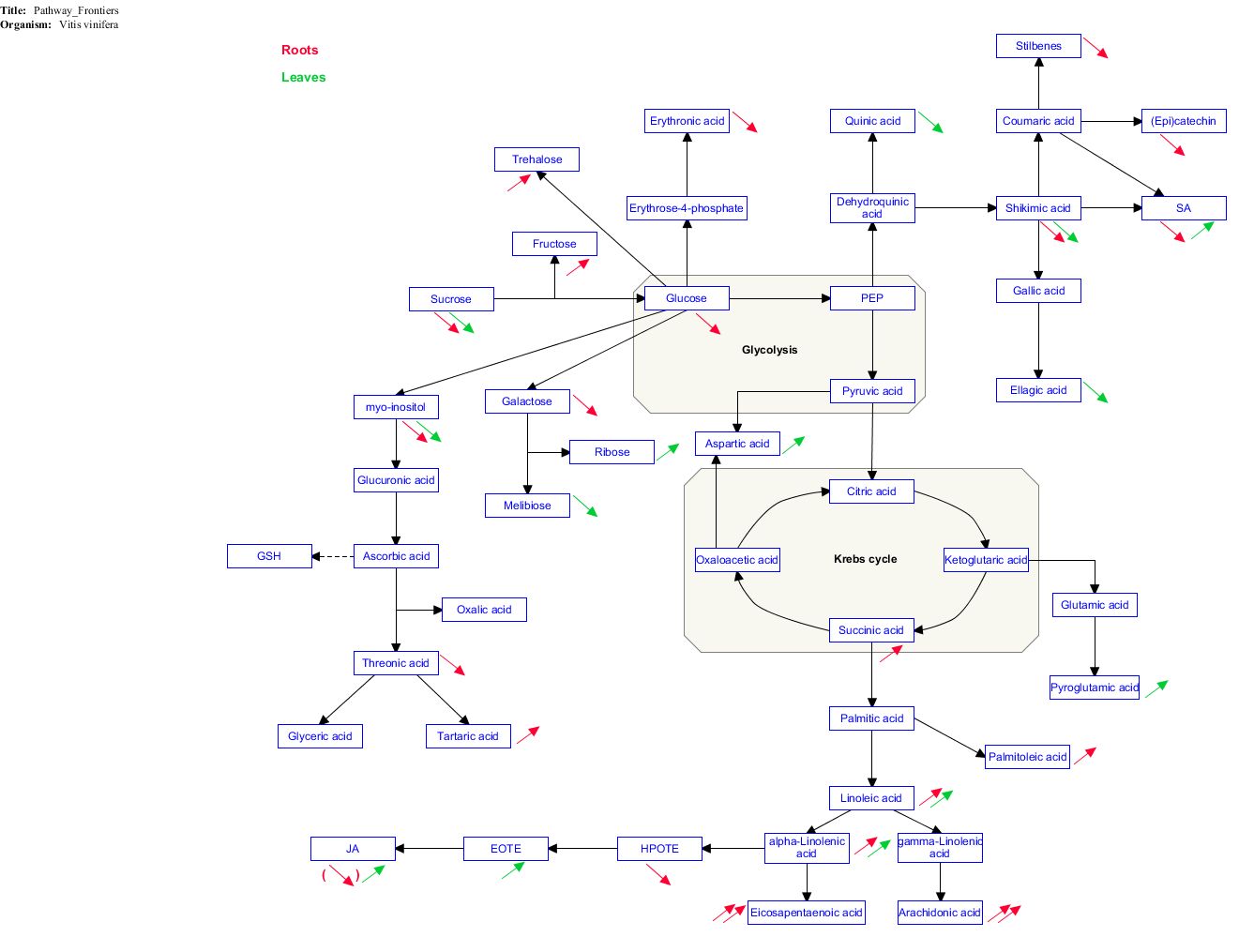
**

Arrows indicate significant increase or decrease in roots (red) or leaves (green). Red Double arrows indicate detection only in Ri roots. Figure was drawn with PathVisio 3.
