# Supplementary material for: Arbuscular Mycorrhizal Symbiosis Triggers Major Changes in Primary Metabolism Together With Modification of Defense Responses and Signaling in Both Roots and Leaves of Vitis vinifera
Source: Front Plant Sci. 2021 Aug 25;12:721614. doi: 10.3389/fpls.2021.721614 (PMC8424087; doi:10.3389/fpls.2021.721614)
Supplement: Supplementary Figures 1–4 — Three-dimensional principal component analysis (3D PCA) of GC-MS and LC-MS metabolite levels in roots and leaves. [file Data_Sheet_1.zip › Supplementary Tables S1-S2.DOCX]

**Supplementary Table 1. Primers used in this study**

| **Gene** | **Accession number (Genbank)** | **Forward Primer 5’ 🡪 3’** | **Reverse Primer 5’ 🡪 3’** |
| --- | --- | --- | --- |
| *VvACT1* | AF369524^a^ | TGCTATCCTTCGTCTTGACCTTG | GGACTTCTGGACAACGGAATCTC |
| *VvEF1α* | CB977561^a^ | GAACTGGGTGCTTGATAGGC | AACCAAAATATCCGGAGTAAAAGA |
| *VvLOX3* | NM_001281249.1^a^ | GGACCGGGTTCATGAGCTGTTGG | TGAATGCAGACTCGCCAGCGGT |
| *VvLOX9* | AY159556.1^a^ | GACAAGAAGGACGAGCCTTG | CATAAGGGTACTGCCCGAAA |
| *VvAOS1* | XM_002281190.3^a^ | TTATGGCTTGCCCTTCTTTGG | ATGGAGTCGAGGAGGACGAT |
| *VvAOC1* | XM_002273394.3^a^ | AGCCAGCCAACTCACTGG | AGCGGTCACCCTTCCTCTCC |
| *VvSUC11* | XM_019226711.1^a^ | GTCGCCGTTCTGATAATC | ATTAGCCACATCCAATAACC |
| *VvSUC12* | XM_010653514.2 ^a^ | GCAGGCTGAGGTTGAATTG | GTGGATGAGGAATGGATGAAG |
| *VvSUC27* | NM_001281141.3 ^a^ | CGTTATCTCTGTTCGCTGTTATG | GCTACCGACACCATCATCTG |
| *VvSWEET4* | GSVIVT01032489001^b^ | GGCTCGGACTGTGATTGGTA | ACATGCAGTTCATCACTGTGG |
| *VvSWEET12* | GSVIVT01008597001^b^ | GGCTACTTCTTCTAATAAACCTTGGAG | GAATACGCTCACAGCGAATATGA |
| *VvSWEET17c* | GSVIVT01031172001^b^ | AGCTTGGAAGGTGTTAGTGTGAT | CATGGAACACACACATTGAGG |
| *VvHT5* | NM_001281278.1 ^a^ | GTCGCTTGGAAGAAGGAAAG | CCTACTTTGTCGACAGAGTAGACG |
| *VvCIN2* | XM_010649021.2 ^a^ | TGGAGGACCCTTCCCATCTG | TTGGAATTTGGAAGAAGTGTGTTC |
| *VvWINV* | NM_001281279.1 ^a^ | ACGAATCATCTAGTGTGGAGCAC | CTTAAACGATATCTCCACATCTGC |
| *VvWRKY2* | AY596466.2 ^a^ | AGAGGCAAGGCGATGTAGAA | CTGGGGAACAAGCCTTCATA |
| *VvPR6 bis* | XM_019219942.1^a^ | GCCAGAGCTGGTGGGCGTAC | AGGCGCCATACTCACGATGCC |
| *VvPR7* | XM_019217396.1 ^a^ | TGCTCCCAATCATGGTGGCTGT | TGAAGACTCTGCGGTGTGTCCT |
| *VvPR7 bis* | XM_019217401.1 ^a^ | CGTTAAGCAGCTGGAAAGGAGCA | TCCTCCGTCAGTCTGGCTGCAA |
| *RiMST2* | HM143864.1 ^a^ | GGCAGGATATTTGTCTGATAG | GCAATAACTCTTCCCGTATAC |
| *VvPht 1.2* | VIT_16s0050g02370 ^b^ | CGTGAGGCGGATTTTCTGT | ATCAAAGAACTCTCTCGACCAT |

^a^  NCBI GenBank accession number

^b^ Genoscope accession number (http://www.genoscope.cns.fr/externe/GenomeBrowser/Vitis/)

**Supplementary Table S2. Jasmonates quantification. Target monitored ions, chromatographic retention times (RT), assigned internal standards, quantifier and qualifier ions**

| **Compound** | **Formula** | **RT (min)** | **Internal standard** | **Quantifier m/z** | **Qualifier m/z** |
| --- | --- | --- | --- | --- | --- |
| JA | C_12_H_18_O_3_ | 13.0 | dh-JA-Ile | 209.1183 | 59.0139 |
| JA-Ile | C_18_H_29_NO_4_ | 14.5 | dh-JA-Ile | 322.2024 | 130.0874 |
| 12-OPDA | C_18_H_28_O_3_ | 16.2 | PGA1 | 291.1966 | 165.1285/247.2067 |
| SA | C_7_H_6_O_3_ | 11.3 | dh-JA-Ile | 137.0244 | 93.0346 |
